# Synthetic (*E*)-3-Phenyl-5-(phenylamino)-2-styryl-1,3,4-thiadiazol-3-ium Chloride Derivatives as Promising Chemotherapy Agents on Cell Lines Infected with HTLV-1

**DOI:** 10.3390/molecules25112537

**Published:** 2020-05-29

**Authors:** Danilo Sousa-Pereira, Thais Silva de Oliveira, Rojane O. Paiva, Otávio Augusto Chaves, José C. Netto-Ferreira, Juliana Echevarria-Lima, Aurea Echevarria

**Affiliations:** 1 Instituto de Química, Universidade Federal Rural do Rio de Janeiro (UFRRJ), Seropédica, Rio de Janeiro 23.890-000, Brazil; sousadanilo90@gmail.com (D.S.-P.); otavioaugustochaves@gmail.com (O.A.C.); 2 Laboratório de Imunologia Básica e Aplicada, Departamento de Imunologia, Instituto de Microbiologia Paulo de Góes, Universidade Federal do Rio de Janeiro (UFRJ), Rio de Janeiro 21.941-590, Brazil; thais.silvadeoliveira@yahoo.com.br (T.S.d.O.); rojanedeoliveirapaiva@gmail.com (R.O.P.); 3 Instituto SENAI de Inovação em Química Verde (ISI QV), Maracanã, Rio de Janeiro 20.271-030, Brazil; 4 Instituto Nacional de Metrologia, Qualidade e Tecnologia (INMETRO), Divisão de Metrologia Química, Duque de Caxias, Rio de Janeiro 25.250-020, Brazil

**Keywords:** mesoionic compounds, HTLV-1, HSA interaction, multiple spectroscopic, molecular docking

## Abstract

Synthesis of four compounds belonging to mesoionic class, (*E*)-3-phenyl-5-(phenylamino)-2-styryl-1,3,4-thiadiazol-3-ium chloride derivatives (**5a**–**d**) and their biological evaluation against MT2 and C92 cell lines infected with human T-cell lymphotropic virus type-1 (HTLV-1), which causes adult T-cell leukemia/lymphoma (ATLL), and non-infected cell lines (Jurkat) are reported. The compounds were obtained by convergent synthesis under microwave irradiation and the cytotoxicity was evaluated using 3-(4,5-dimethylthiazol-2-yl)-2,5-diphenyltetrazolium bromide (MTT) assays. Results showed IC_50_ values of all compounds in the range of 1.51–7.70 μM in HTLV-1-infected and non-infected cells. Furthermore, it was observed that **5b** could induce necrosis after 24 h for Jurkat and MT2 cell lines. The experimental (fluorimetric method) and theoretical (molecular docking) results suggested that the mechanism of action for **5b** could be related to its capacity to intercalate into DNA. Moreover, the preliminary pharmacokinetic profile of the studied compounds (**5a**–**d**) was obtained through human serum albumin (HSA) binding affinity using multiple spectroscopic techniques (circular dichroism, steady-state and time-resolved fluorescence), zeta potential and molecular docking calculations. The interaction HSA:**5a**–**d** is spontaneous and moderate (*K_a_* ~ 10^4^ M^−1^) via a ground-state association, without significantly perturbing both the secondary and surface structures of the albumin in the subdomain IIA (site I), indicating feasible biodistribution in the human bloodstream.

## 1. Introduction

In 2018, Global Cancer Statistics estimated that 18.1 million new cases of cancer and 9.6 million deaths (excluding nonmelanoma skin cancer) would occur around the world. Among these new cases, it was estimated that the incidence of leukemia and lymphoma (non-Hodgkin) would be approximately 1 million cases worldwide [1]. Leukemia was characterized by substantial impairment of bone marrow and the presence of circulating tumor cells in peripheral blood. Leukemia can be classified based on disease progression and related cell lines [2]. Human T-cell lymphotropic virus type 1 (HTLV-1) is an etiological agent of the adult T-cell leukemia/lymphoma (ATLL) malignancy [3] and the HTLV-1-associated myelopathy/tropical spastic paraparesis (HAM/TSP), an inflammatory disease [4]. The viral particles and reverse transcriptase enzyme were identified from a peripheral blood sample from a T-cell leukemia patient, leading HTLV-1 to be described as the first neoplastic-associated retrovirus in humans [5]. It is estimated that 10 to 20 million individuals worldwide are infected with HTLV-1. The HTLV-1 infection is higher prevalent in Japan, Africa, and Central and South America [6]. On the other hand, Japan has the highest prevalence of ATLL in the world and 1000 new cases are diagnosed annually [7].

The predominant target of HTLV-1 infection is the CD4^+^ T lymphocyte, which induces activation and leads to spontaneous proliferation of these cells [8]. Cell proliferation induces genomic instability, leading to chromosomal abnormalities, culminating in cell transformation [9]. Unfortunately, treatment with ATLL is restricted and patients with aggressive disease have poor prognosis due to the state of immunosuppression and resistance of the leukemic cells to multiple drugs. ATLL is classified into four subtypes, based on clinical differences and prognostic factors: acute, lymphoma, chronic, and smoldering [10,11,12]. Acute or lymphoma subtypes represent 60% of ATLL types, with a one-year prognosis of median survival, approximately [13]. The clinical features are heterogeneous lymphadenopathy, splenomegaly, hepatomegaly, hypercalcemia, skin and lung lesions, and lymphocyte infiltration [12].

**Figure 1:**
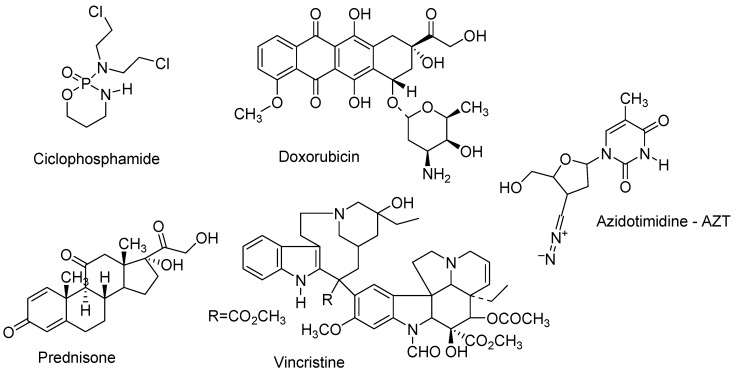
Chemical structures for the chemical compounds that make up the CHOP cocktail (cyclophosphamide, doxorubicin, prednisone, and vincristine) and AZT (ziduvidine) used in adult T-cell leukemia/lymphoma (ATLL) chemotherapy.

The literature reports several treatment protocols for ATLL with variable results. The treatment strategy depends on ATLL subtype. Some protocols employ chemotherapy, with the use of ziduvidine (AZT)/interferon-α (IFN-α) and hematopoietic stem cell transplantation, which may occur in association or not depending on the aggressiveness of the disease [14]. The AZT/IFN-α association allowed good results, considering the acute, chronic, and indolent forms of ATLL, since the immunological effect of IFN-α is associated with the cytostatic effect of AZT on lymphoid cells [15]. The most-used treatment in the face of the lymphomatous and acute clinical forms of ATLL (more aggressive) is the use of the CHOP cocktail: (C) cyclophosphamide; (H) hydroxyldaunorubicin (doxorubicin); (O) oncovin (vincristine); and (P) prednisone. Cyclophosphamide is an alkylating agent that causes the formation of cross-links with deoxyribonucleic acid (DNA), hydroxyldaunorubicin damages DNA by inserting itself between the bases (intercalating agent), oncovin prevents cell duplication through binding to tubulin protein, and prednisone acts on cytokines (inflammatory process) [13]. Figure 1 shows the structures for CHOP and AZT chemotherapeutic drugs.

The AZT/IFN-α and CHOP combination showed more prolonged survival in patients when compared to chemotherapy alone. However, resistance to conventional chemotherapy, the increase in proteins responsible for resistance to multiple drugs (MDR—multiple drugs resistance), hepatic or renal dysfunction, and the state of immunodeficiency during chemotherapy are still factors associated with failure in the treatments used. In addition, the long latency of HTLV-1 infection may contribute to the failure of ATLL therapy [13,16,17]. Moreover, ATLL trial treatments using mogamulizumab (anti-CCR4 monoclonal antibody) or/and lenalidomide ((*RS*)-3-(4-amino-1-oxo-1,3-dihydro-2*H*-isoindol-2-yl)piperidine-2,6-dione), a thalidomide derivate, were employed in Japan [18]. Mogamulizumab was also used in combination with multi-agent chemotherapy, improving the therapy response of 33% to 52% [19].

Heterocyclic compounds of the mesoionic class, especially those with 1,3,4-thiadiazole moiety, have been synthesized and their biological effects investigated, confirming their excellent potential biological activity. Different biological activities have been described for these compounds, such as anticancer [20,21,22,23], antiparasitic [24,25,26], anti-inflammatory, analgesic [27,28], and antimicrobial [29,30]. Mesoionic compounds have a betaine-like characteristic, with a positive charge on the heterocyclic ring and a negative charge on the exocyclic atom and, therefore, can be represented only by dipolar structures [31].

**Scheme 1:**
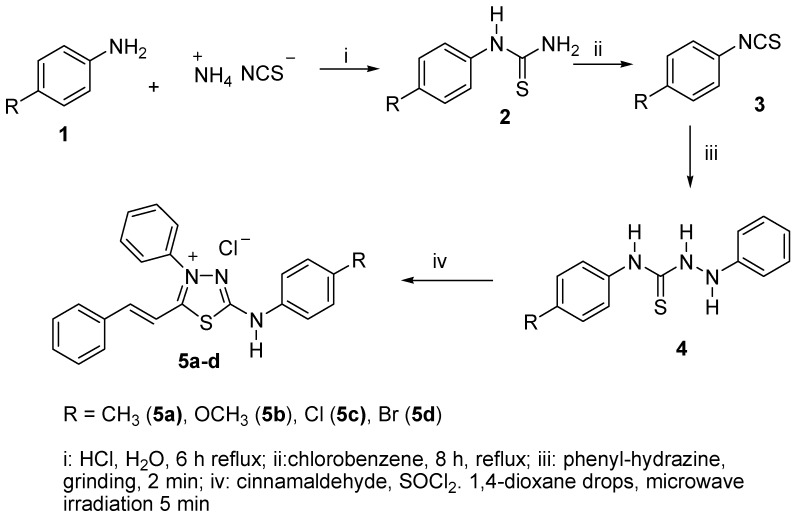
Synthetic route for obtaining the target compounds **5a–d**.

In our previous studies with 1,3,4-thiadiazolium salts (mesoionic compounds) containing a substituted styryl moiety, important results for in vitro cytotoxic activity in various types of tumor cells, including leukemias [23,32,33,34], and in vivo antitumor effects have been observed [35,36]. Besides, results on the antiviral activity of 1,3,4-thiadiazole derivatives against HIV-1 [37,38,39,40], which also belong to the same Retroviridae family, have also been reported in the literature. These results motivated us to synthesize new 1,3,4-thiadiazolium salts containing an unsubstituted styryl group, as well as a substituted phenyl ring linked to the exocyclic nitrogen (Scheme 1). 

Human serum albumin (HSA), one of major proteins in the human blood circulation system, takes part in many physiological functions. Its proportion in plasma proteins is about 55% [41]. HSA has a remarkable capacity for reversible binding with drugs, which helps in improving their solubility, protect their oxidation in plasma, and decrease toxicity. Because of these exceptional abilities, HSA is one of the key targets to predict the pharmacokinetic profile for development of a potential therapeutic agent [42]. From the structural point of view, HSA is a non-glycosylated heart-shaped single chain polypeptide with molecular weight of 66.5 kDa (80 × 80 × 30 Å in size), being divided into three analogous domains (I, II, and III) having amino acid residues of 1–195, 196–383, and 384–585, respectively [43]. Each domain comprises two subdomains (A and B) and all domains are balanced through a bridge of 17-disulfide bonding. HSA has two main hydrophobic cavities for ligand binding: subdomain IIA (site I) and subdomain IIIA (site II) [44]. The main fluorophore of HSA is the tryptophan (Trp-214) residue, which is located in a large hydrophobic cavity in subdomain IIA (site I) [45]. Therefore, the intrinsic fluorescence intensity of Trp-214 residue is a common phenomenon used to investigate the interaction of ligands with HSA [46,47,48].

Based on the importance of 1,3,4-thiadiazolium salts in the medicinal chemistry, the present study reports the synthesis and characterization of four heterocyclic compounds, namely (*E*)-3-phenyl-5-(phenylamino)-2-styryl-1,3,4-thiadiazol-3-ium chloride derivatives, where R = CH_3_ (**5a**), OCH_3_ (**5b**), Cl (**5c**), and Br (**5d**). Each compound was assayed against C91 and MT2 cell lines infected with HTLV-1 and a Jurkat cell line (lymphocytic leukemia). In addition, it was also investigated how the mesoionic compounds **5a**–**d** induce apoptotic cell death, as well as their preliminary pharmacokinetic profile via affinity for HSA by multiple spectroscopic techniques combined with molecular docking. It is important to highlight that the synthesis of the mesoionic compounds **5a**, **5b**, and **5d** have already been described [29] (**5c** is a new synthetic mesoionic), however, this work reports for the first time the evaluation of **5a**–**d** as promising chemotherapy agents.

## 2. Results and Discussion

### 2.1. Synthesis

The compounds (*E*)-3-phenyl-5-(phenylamino)-2-styryl-1,3,4-thiadiazol-3-ium chloride derivatives (**5a–d**) were prepared following Scheme 1 as previously reported [23] using microwave irradiation as the power supply. Initially, the aryl-thioureas were prepared from substituted anilines and ammonium isothiocyanate in the presence of concentrated HCl and water as solvent. The aryl-thioureas obtained (**2a–d**) were submitted under 8 h of reflux in chlorobenzene as solvent, and the aryl-isothiocyanates (**3a–d**) were used immediately in the subsequent step as crude products [49] to obtain the *N*-aryl-2-phenyl-hydrazinecarbothioamides (**4a–d**). The intermediates **4a–d** were prepared in solvent free conditions using a grinding method with aryl-isothiocyanates and phenyl-hydrazine at room temperature for 2 min. These compounds were obtained in quantitative yields and high purity, and their structures were confirmed by FT-IR spectroscopy and NMR ^1^H and ^13^C spectrometry, in accordance with previous publications on mesoionic compounds [29,50].

The (*E*)-3-phenyl-5-(phenylamino)-2-styryl-1,3,4-thiadiazol-3-ium chloride derivatives (**5a–d**) were prepared by reaction of cinnamaldehyde and the *N*-aryl-2-phenyl-hydrazinecarbothioamides (**4a–d**) in equimolar amounts, thionyl chloride (excess of three times), and enough drops of 1,4-dioxane to homogenize the reagents. The mixture was submitted to microwave irradiation for 5 min and then poured into 1,4-dioxane. After standing for 24 h, the products were isolated and washed thoroughly with ice water and obtained in good yields (96% to 98%). The compounds **5a–d** were characterized by FT-IR, ^1^H, and ^13^C-NMR spectroscopies (Figures S1–S4 in the Supplementary Material), and their purity was confirmed by elemental analysis. The infrared spectra showed the characteristic absorptions in the range of 3431–3433 cm^−1^ assigned to C=N, 2717 cm^−1^ and 2746 cm^−1^ to **5b** and **5a**, respectively, and 2586 cm^−1^ and 2663 cm^−1^ to **5c** and **5d**, respectively, attributed to C=NH^+^ (stretching), according to other compounds of this class [23]. In the ^1^H-NMR spectra the chemical shift attributed to the exocyclic amino hydrogen was in the range of 12.58 to 12.86 ppm with a singlet feature. The signals of H-6 were assigned at 7.04–7.24 ppm, and of H-7 at 8.02–8.10 ppm, as doublets (*J* = 14 Hz). In the ^13^C-NMR spectra the characteristic signals of heterocyclic carbons were attributed to C-2 at 128.5 ppm (**5a**), 156.0 ppm (**5b**), 158.8 ppm (**5c**), and 158.9 ppm (**5d**), and to C-5 159.8 ppm (**5a**), 161.3 ppm (**5b**), 163.2 ppm (**5c**), and 163.4 ppm (**5d**). The carbon atoms C-6 and C-7 showed signals coherent with the transmission of the electronic effect of substituents in the *N*-X-phenylamine group linked to the heterocyclic ring, that is, the C-6 had more shielding in 111.5 ppm to 115.5 ppm and C-7 had more deshielding in 142.8 ppm to 148.2 ppm. The other carbon atoms appeared with the chemical shifts as expected according to the literature [15].

### 2.2. Biological Activity 

**Figure 2:**
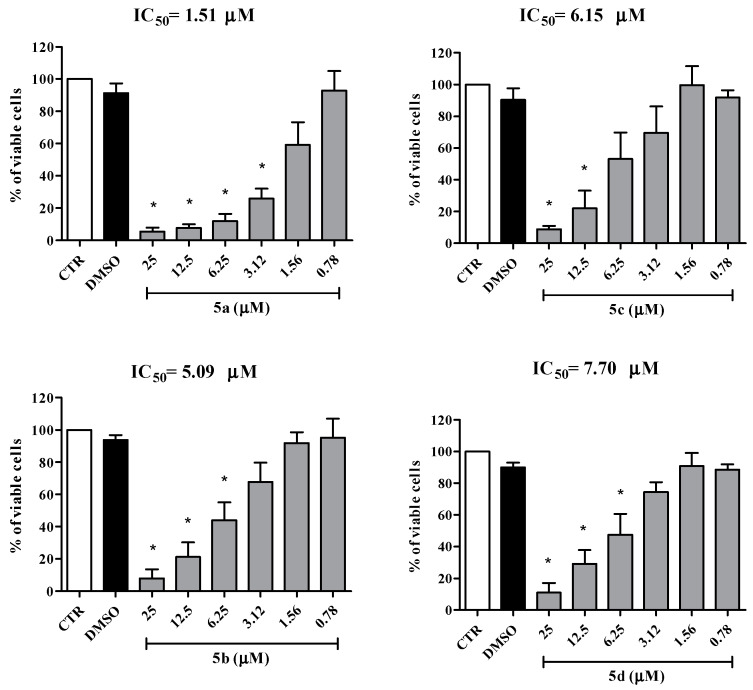
Effect of mesoionic compounds **5a–d** on MT2 cells. The HTLV-1-infected cell line, i.e., MT2, was incubated in the presence or absence of **5a–d** (0.78–25 µM) at 37 °C in a humid atmosphere containing 5% CO_2_. After 72 h, the cells were incubated with MTT for 3 h under the same conditions. Then, cells were centrifuged, and DMSO was added to dissolve the crystals formed by reducing the MTT. The bars represent mean ± SEM values of % of viable cells from 4 to 5 independent experiments. * *p* < 0.05 were considered statistically significant compared to control and DMSO.

**Figure 3:**
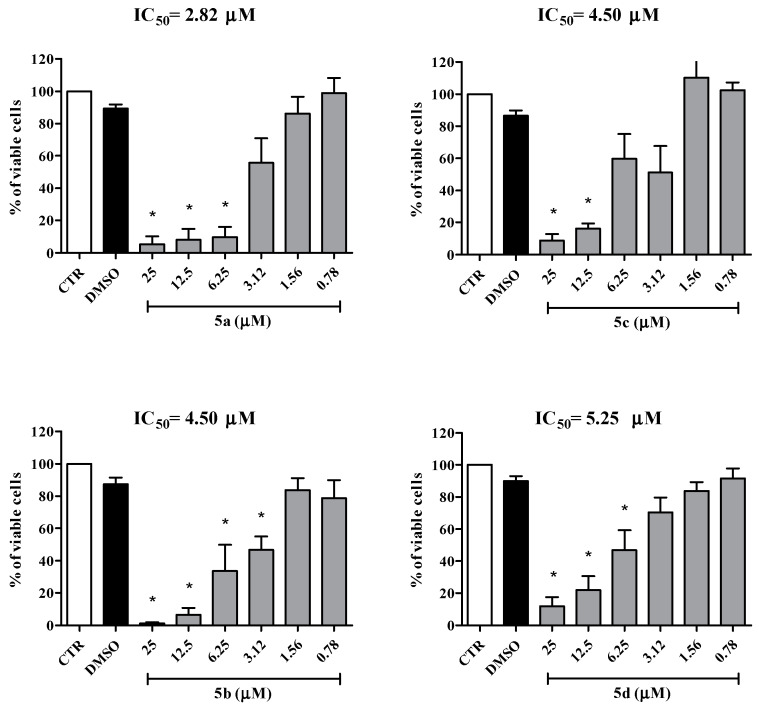
Effect of mesoionic compounds **5a–d** on HTLV-1-infected cells. C91/PL cells were incubated in the presence or absence of **5a–d** (0.78–25 µM) at 37 °C in a humid atmosphere containing 5% CO_2_. After 72 h, the cells were incubated with MTT for 3 h under the same conditions. Then, cells were centrifuged, and DMSO was added to dissolve the crystals formed by reducing the MTT. The bars represent mean ± SEM values of % of viable cells from 4 to 7 independent experiments. * *p* < 0.05 were considered statistically significant compared to control and DMSO.

**Figure 4:**
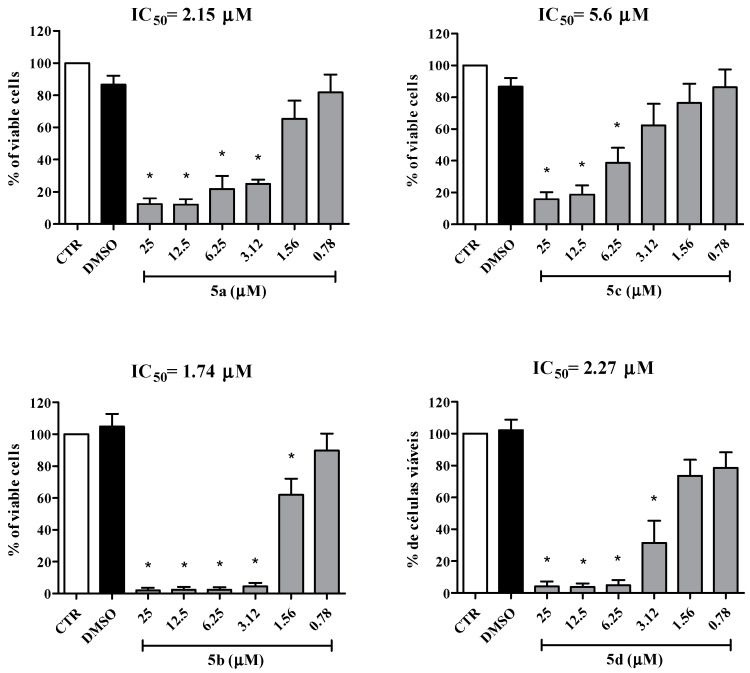
Effect of mesoionic compounds **5a–d** on Jurkat cells. Cells were incubated in the presence or absence of **5a–d** (0.78–25 µM) at 37 °C in a humid atmosphere containing 5% CO_2_. After 72 h, the cells were incubated with MTT for 3 h under the same conditions. Then, cells were centrifuged and DMSO was added to dissolve the crystals formed by the MTT reduction. Bars represent mean ± SEM values of % of viable cells from 4 to 5 independent experiments. * *p* < 0.05 were considered statistically significant compared to control and DMSO.

**Figure 5:**
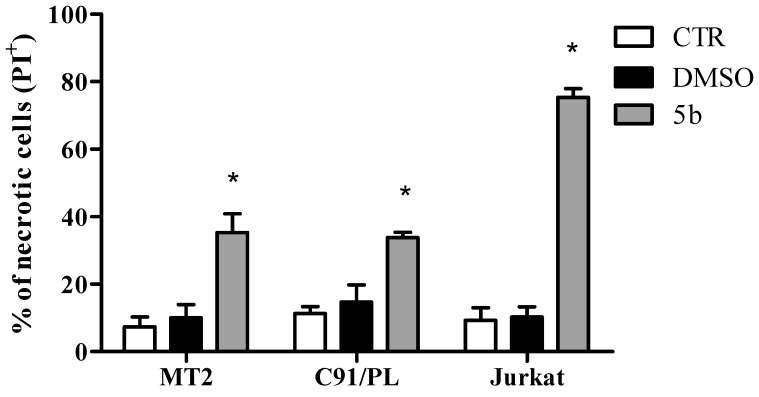
Effect of compound **5b** on induction of necrosis in HTLV-1 infected MT2, C91/PL, and Jurkat cells. HTLV-1-infected cells and Jurkat cells were incubated with or without the compound **5b** (25 µM) at 37 °C in a humid atmosphere containing 5% CO_2_. After 24 h, the cells were collected, and the cell viability was measured using PI (propidium iodide) labeling. Then, the fluorescence was examined using flow cytometry. A total of 10,000 events was acquired for the experimental data. Bars represent mean ± SEM values of % of necrotic cells from three independent experiments. * *p* < 0.05 were considered statistically significant compared to control and DMSO.

The anti-leukemia activity of mesoionic compounds has been investigated since the 1970s [51], with their anti-leukemia effects having been described both in vivo and in vitro [51,52,53]. However, the cytotoxicity effect of this class of compounds on cells infected with HTLV-1 has not yet been evaluated. In the present work, it was demonstrated for the first time that the cell lines (MT2 and C91/PL) infected with HTLV-1 and treated with mesoionic compounds **5a–d** significantly reduced cell viability (Figure 2 and Figure 3). In addition, **5a–d** also exhibited a cytotoxicity effect against Jurkat cells, an uninfected cell line in adult T leukemia (Figure 2). The results obtained demonstrated that treatment with these mesoionic compounds reduced cell viability in a dose-dependent manner (Figure 2, Figure 3 and Figure 4). From Figure 5 it can be seen that **5b** was able to induce necrosis in Jurkat cells (75.37 ± 3.63%), MT2 (35.34 ± 7.80%), and C91/PL (33.75 ± 1.65%) after 24 h of cell treatment.

Each cell line showed different sensitivity to the **5a–d** mesoionic compounds, as evidenced by the IC_50_ values shown in Table 1. Interestingly, our results indicated that the values obtained for IC_50_ were less than 10 µM. Furthermore, these results clearly show that compound **5a** has the highest cytotoxicity value against HTLV-1 infected cell lines, i.e., MT2 or C91/PL, with IC_50_ values of 1.51 and 2.82 µM, respectively. On the other hand, compound **5b** showed significant inhibitory activity in Jurkat cells with an IC_50_ value of 1.74 µM. It is important to note that the mesoionic compounds with greater activity, **5a** (R = CH_3_) and **5b** (R = OCH_3_), have a substituent group displaying an electron donor property, which suggests that this characteristic may be related to the results of cytotoxicity. In addition, values of log Po/w of 3.74 and 3.35 for **5a** (R = CH_3_) and **5b** (R = OCH_3_), respectively, indicate that these compounds are less hydrophobic than compounds **5c** (R = Cl) and **5d** (R = Br), for which log Po/w are 3.97 and 4.10, respectively. In the literature, several authors have suggested that reduced hydrophobicity can be associated with the efficiency of cytotoxicity, as this property is directly related to the binding to cellular targets [54,55]. Besides, retrovirus infection alters lipid synthesis, leading to an alteration in the cell membrane [56]. The HTLV-1 genome has a pX region, which encodes viral accessory genes, such as tax. The transcription of the tax gene is a Tax protein responsible for modifying or decreasing the modulation of many cellular genes, interfering with cell proliferation, apoptosis, secretion, and others [57]. Thus, the difference observed between the effect of cytotoxicity in cells infected with HTLV-1 and Jurkat cells may be directly related to viral modulation in the synthesis of lipids and proteins.

**Figure 6:**
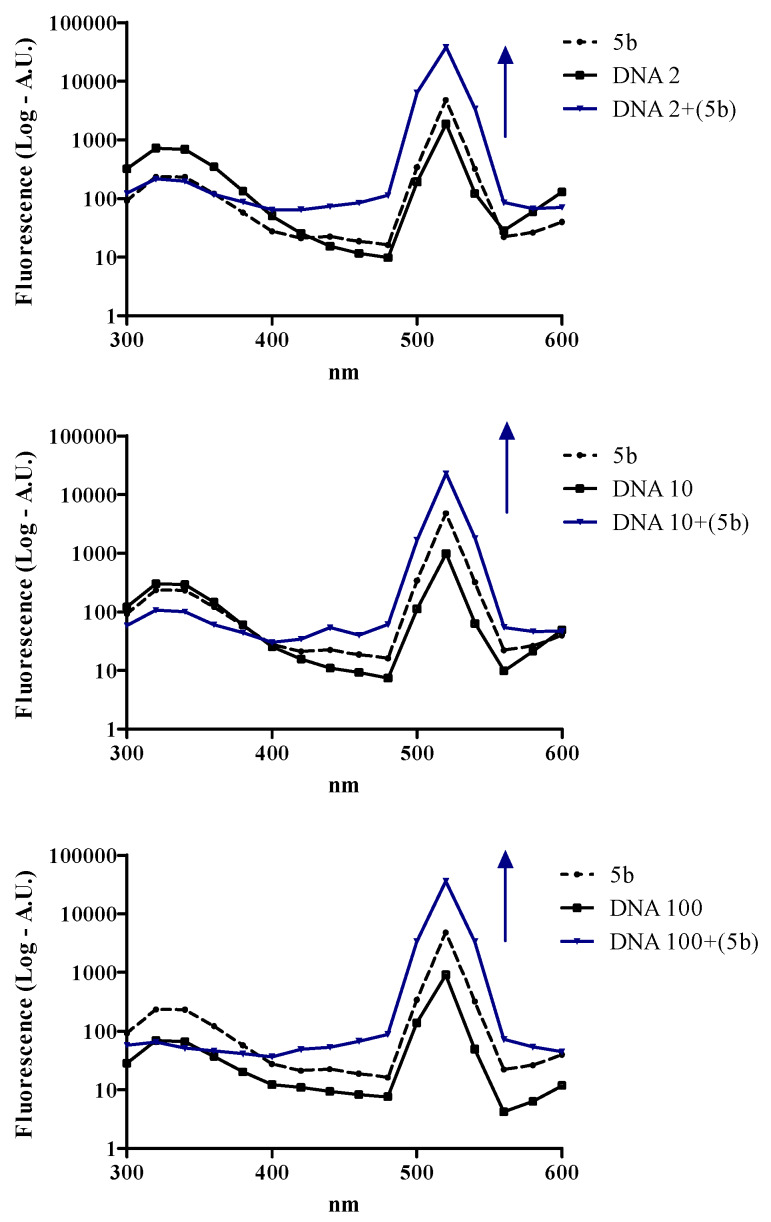
Fluorescence emission spectra of **5b** (25 μM) in the presence of increasing amounts of DNA (2, 10, and 100 ng/mL). A.U. = arbitrary unit.

It is known that mesoionic compounds can interact with the DNA molecule and their biological activity can be related with this interaction [32,58]. Based on this, the interaction of compounds **5a–d** with DNA was investigated using spectroscopic analysis. The fluorescence emission spectra of pure DNA and the corresponding DNA complex with the mesoionic compounds under study are shown in Figure 6 for **5b,** whereas Figure S5 in the Supplementary Material shows the DNA assays for **5a**, **5c**, and **5d**. The experimental results suggested that only compound **5b** can interact with the DNA strands; however, the mechanism of this interaction remains unknown, and more tests are needed to indicate its nature.

**Figure 7:**
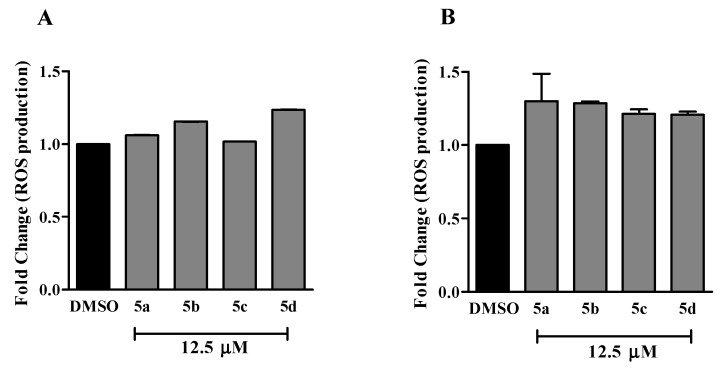
Effect of mesoionic compounds **5a–d** on reactive oxygen species (ROS) production. Jurkat (**A**) and MT2 (**B**) cells were incubated with or without **5a–d** (12.5 µM) at 37 °C in a humid atmosphere containing 5% CO_2_. After 30 min, the cells were washed and incubated with the ROS probe DHR (Dihydrorhodamine 123). Then, the fluorescence was examined using flow cytometry. A total of 10,000 events was acquired for the experimental data. Bars represent mean ± fold change of ROS production (DMSO was considered as a control) from two independent experiments.

Some anticancer drugs approved by the FDA (US Agency for Food and Drug Administration) can induce cytotoxicity effects through reactive oxygen species (ROS) modulation, including antileukemia drugs, such as L-asparaginase, *N*-benzyloxycarbonyl-IIe-Glu-(*O*-t-butyl)-Ala-leucinal, anthracyclines (doxorubicin and daunorubicin), arsenic trioxide, and 2-methoxyestradiol. These drugs diminished the reduced glutathione intracellular levels and induced mitochondrial dysfunction or ROS generation by other pathways [59]. Thus, the capacity of mesoionic compounds **5a–d** to produce ROS was evaluated. Using the fluorescent dye dihydrorhodamine 123 (DHR), it was observed that a short treatment (30 min) of the cells (MT2 and Jurkat) with these compounds induced an increase in ROS levels, indicating that the oxidative stress can be a mechanism of action of these substances (Figure 7). These mesoionic compounds can also induce nitric oxide production, which inhibits cellular proliferation and promotes cytostatic action [53,60]. Overall, our results suggest that the mesoionic compounds **5a–d** constitute a promising class of new anticancer drugs.

### 2.3. Interaction with HSA

#### 2.3.1. Experimental Binding Ability

The steady-state fluorescence spectra for HSA (1.00 × 10^−5^ M) in the absence and presence of successive additions of mesoionic compounds **5a–d** (concentration up to 1.32 × 10^−5^ M) are shown in Figure S6 in the Supplementary Material. In the presence of these compounds, it is possible to observe the quenching of the fluorescence emission of the protein (λ_em_ = 340 nm), which indicates the existence of a possible interaction between the Trp-214 residue, responsible for the emission of albumin, and **5a–d** [50,61]. The addition of any of these mesoionic compounds to the HSA solution did not result in a shift in the maximum fluorescence emission, indicating that the HSA:mesoionic compound interaction does not disturb the microenvironment surrounding the Trp-214 residue [46].

The steady-state fluorescence quenching of a protein can be classified as static and/or dynamic [62]. A Stern–Volmer analysis of the HSA quenching process for **5a–d** (Figure S7 in the Supplementary Material) shows values for the bimolecular quenching rate constant of the order of *k_q_* ~ 10^13^ M^−1^s^−1^ (Table 2). These values are about four orders of magnitude higher than for the water limiting diffusion rate constant (*k_diff_* ~ 7.40 × 10^9^ M^−1^s^−1^, at 298K [63]). This is an indication that the fluorescence quenching mechanism must be static, involving an association in the ground-state between albumin and mesoionic compounds **5a–d** [50]. In addition, the values for the Stern–Volmer constant (*K_SV_*) at different temperatures are similar (Table 2), suggesting that **5a–d** show similar binding capacity towards HSA.

To further confirm which mechanism is operating in the fluorescence quenching of HSA by mesoionic compounds **5a–d**, time-resolved fluorescence measurements were performed. The fluorescence decay for HSA is bi-exponential, with lifetimes of *τ_1_* = 1.73 ± 0.16 ns and *τ_2_* = 5.54 ± 0.12 ns (Figure S8, Supplementary Material), which is in accordance with literature data [64,65]. Both fluorescence lifetimes are independent of the addition of any of the mesoionic compounds **5a–d** (1.35 × 10^−5^ M concentration) (Table 3), which is a clear indication of a static process as the main mechanism fluorescence quenching in the interaction between HSA and the mesoionic compounds under study [47]. Similar results have been reported previously for the interaction between HSA and piperonal mesoionic derivatives [66].

The modified Stern–Volmer binding constant (*K_a_*) is related to the protein–ligand interaction, and its magnitude is important for understanding the distribution of a drug in the human bloodstream [47,61]. Moderate values for *K_a_*, in the order of 10^4^ M^−1^, at 296, 303, and 310 K, were obtained for the interaction HSA:**5a**–**d** (Figure S9 in the Supplementary Material and Table 2), indicating the viability of the absorption and distribution of these mesoionic compounds to various tissues [46,48,61].

The thermodynamic parameters of enthalpy (*ΔH*°) and entropy (*ΔS*°) variation for the interaction between HSA and **5a**–**d** were calculated according to the van’t Hoff approximation (Figure S10, Supplementary Material, and Table 3). The positive values of *ΔS*° for all investigated compounds are related to hydrophobic forces, as a consequence of an interaction between the non-polar amino acid residues and the non-polar portions of the ligand structure. Furthermore, the same hydrophobic effect may also be related to the fact that both the water molecules responsible for the solvation of the ligand and those that occupied the protein cavity are expelled out of the cavity during the HSA:ligand interaction process. In this case, these water molecules present higher increased degrees of freedom and, therefore, an increase in the system disorder (*ΔS*° > 0) [67,68]. 

The *ΔH*° < 0 values for HSA:**5a** and HSA:**5b** indicate a significant contribution of hydrogen bonding, whereas for HSA:**5c** and HSA:**5d**, *ΔH*° > 0 and, in this case, the presence of electrostatic interaction forces can be suggested [67,69].

The negative sign for Gibb’s free energy values (*ΔG*° < 0, Table 2) for HSA:mesoionic compounds is a clear indication of the spontaneity of the binding process. A general analysis of the thermodynamic parameters leads us to conclude that the interaction between HSA:**5a** and HSA:**5b** is enthalpically and entropically driven, while for HSA:**5c** and HSA:**5d** it is only entropically driven [69].

#### 2.3.2. Perturbation on the Surface and Secondary Structure of Albumin

Circular dichroism spectroscopy (CD) is a sensitive technique used to investigate the secondary structure of proteins after binding to a ligand [69]. The CD spectrum for HSA exhibits two negative peaks, at 208 nm and 220 nm, characteristic of the typical α-helix structure of proteins. These two peaks correspond to the π–π * and n–π * transitions, respectively [70]. After adding a solution of the mesoionic compounds **5a–d** (1.32 × 10^−5^ M) to the HSA solution (1.00 × 10^−5^ M), a small decrease in intensity at 208 nm and 222 nm was clearly observed (Figure S11, Supplementary Material), indicating a slight change in the secondary structure of the HSA [50]. The variation in the secondary structure of albumin is about 5.0% in all cases, which is evidence that the **5a–d** interaction can occur with a very slight disturbance in the secondary structure of albumin (Table 4) [71]. The same trend has been reported previously for the interaction between HSA and piperonal mesoionic derivatives [66].

The zeta potential (ZP, ζ) is a useful and feasible parameter for characterizing the surface charge properties of proteins. Changes in the ZP for a protein may imply conformational changes in its structure, modifications on the surface, and/or unfolding or denaturation processes after its binding to the ligand [72]. Table 4 shows the ZP value for HSA without and in the presence of **5a–d**. The ZP values for HSA did not change significantly with the addition of these mesoionic compounds, with this change being within the standard error of the measurement. These results indicated that there is no significant structural change on the surface of the protein after its interaction with **5a–d** [73] and are fully in accordance with the results for circular dichroism described above.

### 2.4. Molecular Docking Evaluation

Molecular docking calculations have become an increasingly important tool for drug discovery, since they can offer a molecular level explanation on the binding ability of potential compounds towards biomacromolecules, as well as evaluate physical-chemical properties related to their biological activity [74]. In this sense, molecular docking calculations were carried out for both DNA and HSA binding ability to evaluate the main binding region and intermolecular forces related between the mesoionic compounds **5a–d** and nucleobases/amino acid residues. 

**Figure 8:**
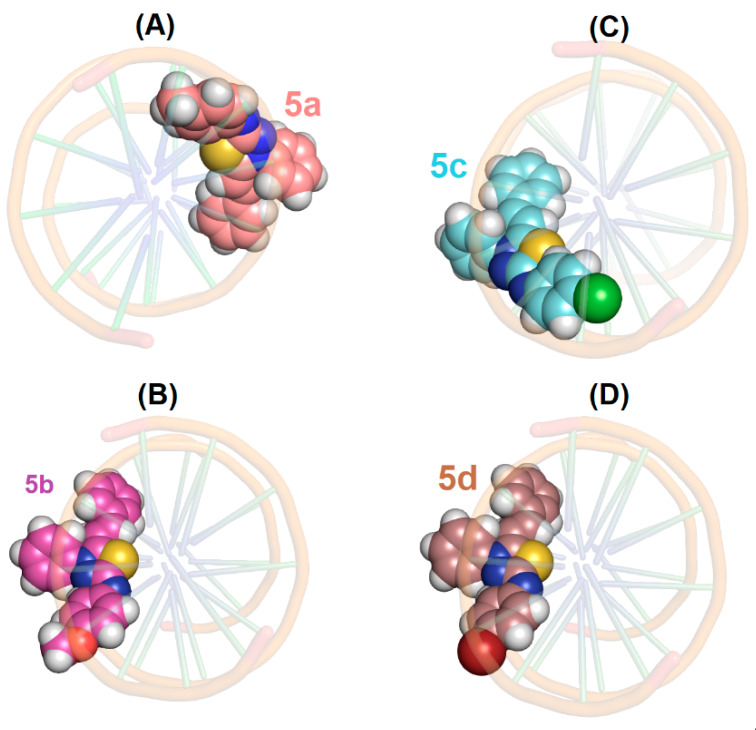
Top view representation for the best docking pose of the interaction (**A**) DNA:**5a**, **(B**) DNA:**5b**, (**C**) DNA**5c**, and (**D**) DNA:**5d** in the minor groove. Mesoionic compounds **5a**, **5b**, **5c**, and **5d** are in stick representation in beige, pink, cyan, and brown, respectively. Elements’ colors: hydrogen: white; oxygen: red; nitrogen: dark blue; sulfur: yellow; bromine: wine; and chloro: green.

As previously described in Section 2.2, it is known that some mesoionic compounds are able to interact with DNA. However, the experimental data indicated that only **5b** could interact with DNA strands. Molecular docking runs for the interaction DNA:**5a–d** offered higher molecular docking score values for the minor groove than for the major groove of DNA strands, i.e., 84.8 and 10.6, respectively for DNA:**5b**, suggesting minor groove as the main possible region of DNA to interact with this mesoionic compound. Differently from the preliminary experimental data, molecular docking results suggested that the mesoionic compounds **5a–d** could interact with DNA, corroborating with the statement that more tests are needed to evaluate the biological mechanism. Figure 8 shows the best docking pose for DNA:**5a–d** via top view representation of the minor groove, reinforcing that the mesoionic compounds can interact with DNA strands mainly via van der Waals interaction (interaction forces were not discussed in the present work).

The HSA structure is composed of three domains (I, II, and III), and each domain is divided into two subdomains (A and B). Pioneering studies by Sudlow using the fluorescent probes displacement method showed the presence of two main specific drug binding sites: Sudlow’s site I, also named the warfarin binding site (subdomain IIA—where Trp-214 residue can be found), and Sudlow’s site II, also named the ibuprofen binding site (subdomain IIIA) [75,76]. Compounds that strongly bind in the Sudlow’s site I are generally bulky heterocyclic molecules with a positive charge localized in its center. On the other hand, Sudlow’s site II is a largely non-polar cavity with a single dominant polar patch near the pocket entrance. This arrangement of polar and non-polar features is consistent with the typical structures of site II drugs, such as aromatic carboxylic acids with a negatively charged acid group at one end of the molecule, separated by a hydrophobic center [77]. Overall, Sudlow’s site II was proposed to be a smaller or narrower site than Sudlow’s site I, because large molecules rarely bind to site II [78]. Recently, subdomain IB, known as site III, was also characterized as a potential binding pocket for different ligands to the albumin structure [47].

**Figure 9:**
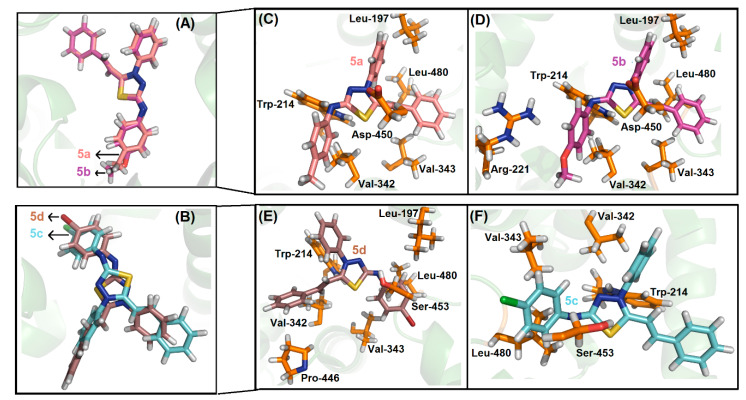
Superposition of the best docking pose for (**A**) HSA:**5a**/**5b** and (**B**) HSA:**5c**/**5d** in the subdomain IIA (site I). Representation of the main amino acid residues from the HSA structure, which can interact with (**C**) **5a**, (**D**) **5b**, (**E**) **5d**, and (**F**) **5c**. Selected amino acid residues and the mesoionic compounds **5a**, **5b**, **5c**, and **5d** are in stick representation in orange, beige, pink, cyan, and brown, respectively. Elements’ colors: hydrogen: white; oxygen: red; nitrogen: dark blue; sulfur: yellow; bromine: wine; and chlorine: green.

Table 5 shows the docking score values for each of the mesoionic compounds **5a–d** to the three main binding sites of HSA. Since the highest docking score value was obtained for site I, molecular docking calculations suggested subdomain IIA, where Trp-214 can be found, as the main binding pocket for the compounds under study. From previous HSA fluorescence quenching studies and the chemical characteristics of each of the mesoionic compounds **5a–d,** it was expected that they should be located next to the amino acid Trp-214 residue in the subdomain IIA. Figure 9 shows the best molecular docking pose for the interaction HSA:**5a–d**. From this Figure, it can be observed that the mesoionic compounds **5a**/**5b** and **5c**/**5d** present a similar docking pose, in accordance with the very close experimental binding parameters obtained from the fluorescence quenching experiments (see Table 2 in Section 2.3.1). In addition, molecular docking results also suggested van der Waals and hydrogen bonding as the main intermolecular forces involved in the binding process, fully in accordance with the experimental data described using the van’t Hoff approximation.

## 3. Materials and Methods 

### 3.1. General Information

Nuclear magnetic resonance (^1^H- and DEPTQ ^13^C-NMR) spectra were recorded on a Bruker Avance III Ultrashield Plus spectrometer (^1^H, 500 MHz; DEPTQ, 125 MHz, Bruker, Rheinstetten, Germany) using DMSO-*d*_6_ as the solvent and tetramethylsilane (TMS) as the internal standard. Fourier-transformed infrared spectra (FT-IR) (ATR) were recorded on a Bruker Vertex 70 spectrophotometer. The elemental analyses were carried out with a Carlo Erba EA-1110 CHNS-O Elemental Analyzer (Elemental Microanalysis, New Jersey, NJ, USA). The melting points were determined on a Gehaka (PF1500 Farma, São Paulo, SP, Brazil) capillary melting point apparatus and uncorrected. The microwave-assisted organic reactions were performed in a CEM Discovery System reactor (CEM Corporation, North Carolina, NC, USA). 

Human acute lymphocytic leukemia (Jurkat cells) were generously provided by Professor Ana Giannini from Universidade Federal do Rio de Janeiro (UFRJ, RJ, Brazil) and HTLV-1 transformed cell lines (MT-2 and C91/Pl) were a gift from the Fundação Oswaldo Cruz, RJ, Brazil. All cells were used and cultured in Roswell Park Memorial Institute (RPMI)-1640 medium supplemented with 10% fetal bovine solution (FBS) (Gibco/Thermo Fisher), 60 mg/mL penicillin and 100 mg/mL streptomycin (LGC, Brazil). They were incubated at 37 °C in a humidified atmosphere of air and 5% CO_2_. The cell line was passaged twice a week.

### 3.2. General Procedure for the Synthesis of Aryl-thioureas (**2a**–**2d**)

The different aryl anilines (23.5 mmol) and ammonium isothiocyanate (23.5 mmol) were mixed in the presence of concentrated hydrochloric acid (23.5 mmol) and 3.17 mL of water as solvent. The reaction mixture was kept under reflux for 6 h. The solid product was obtained by filtration and washed with ice-cold water. All thioureas were identified by the comparison of analytical data (FT-IR, NMR data, and melting points) with literature reports [49].

### 3.3. General Procedure for the Synthesis of Aryl-isothiocyanates (**3a**–**d**)

The aryl-thioureas (7 mmol) was submitted under 8 h of reflux in chlorobenzene as solvent. The solution was then distilled under reduced pressure for chlorobenzene removal. The aryl-isothiocyanates (**3a–d**) were used immediately in the subsequent step as crude products [49]. 

### 3.4. General Procedure for the Preparation of Hydrazinecarbothiamides (**4a**–**d**)

The phenylhydrazine (0.74 mmol) and aryl-isothiocyanate (0.74 mmol) were mixed at room temperature for 2 min in a porcelain mortar and pestle in the absence of any organic solvent. The formed solid was monitored by TLC and was obtained in high purity without recrystallization. All hydrazinecarbothiamides were identified by the comparison of analytical data (FT-IR, NMR data, and melting points) with literature reports [50].

### 3.5. General Procedure for the Preparation of Mesoionic Chlorides (**5a**–**d**)

**Figure 10:**
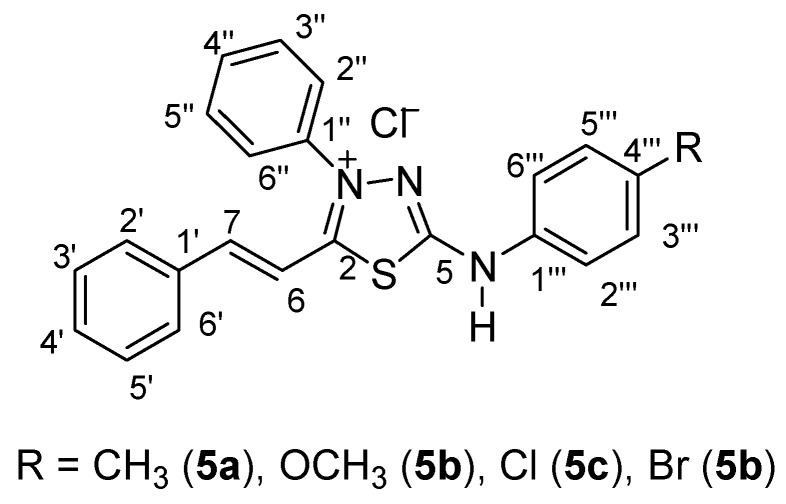
Mesoionic chlorides **5a–d** numbered to the NMR attributions.

The mixture containing the *N*-(4-R-phenyl)-2-phenylhydrazinecarbothioamide (0.38 mmol) and cinnamaldehyde (0.38 mmol) in the presence of SOCl_2_ and a few drops of 1,4-dioxane was submitted to microwave irradiation for 5 min at 100 W. After this continuous time, the mixture was added to 1,4-dioxane and left to stand overnight at room temperature. The solid obtained was filtered and washed with ice-cold 1,4-dioxane and exhaustively with water. The solid formed was obtained in high purity without recrystallization. All mesoionic chlorides (Figure 10) were identified by melting point, FT-IR, NMR data, and elemental analyses [29].

(*E*)-3-Phenyl-5-(4′-methylphenylamino)-2-styryl-1,3,4-thiadiazol-3-ium chloride (**5a**). 

Orange solid; yield 98%; mp 287–286 °C; FT-IR (ATR, υ cm^−1^): 3431 (N-H), 3039 (C-H), 2922 (C-H), 2746 (C=NH^+^), 1614 (C=C), 1564, 1452 (C=C), 1523 (C=N), 1340 (C-S), 1114 (C-H), 823, 763, 688 (C-H); ^1^H-NMR (500 MHz, DMSO-*d*_6_) δ 12.58 (s, 1H, N-H), 8.25 (d, *J* = 7.9 Hz, 2H, H-3′’, H-5′’), 8.10 (d, *J* = 15.9 Hz, 1H, H-7), 8.01 (d, *J* = 9.5 Hz, 2H, H-2′’, H-6′’), 7.83–7.75 (m, 6H, H-2′, H-3′, H-4′, H-5′, H-6′, H-4′’), 7.47 (d, *J* = 7.9 Hz, 2H, H-3′’’, H-5′’’), 7.25 (d, *J* = 15.9 Hz, 1H, H-6), 7.22 (d, *J* = 6.3 Hz, 2H, H-2′’’, H-6′’’), 2.27 (s, 3H, CH_3_); ^13^C-NMR (125 MHz, DMSO-*d*_6_) δ 159.8 (C-2), 148.5 (C-5), 144.5 (C-7), 139.9 (C-1′’’), 136.9 (C-1′), 133.6 (C-1′’), 131.8 (C-4′’), 130.2 (C-2′, C-6′), 130.1 (C-3′, C-5′), 129.9 (C-3′’, C-5′’), 126.1 (C-4′’’), 125.2 (C-2′’, C-6′’), 124.1 (C-3′’’, C-5′’’), 118.8 (C-2′’’, C-6′’’), 115.5 (C-6), 20.5 (CH_3_). Elemental analysis found: C, 67.98%; H, 4.92%; N, 10.41%. Calculated for C_23_H_20_ClN_3_S (MW 405.94): C, 68.05%; H, 4.97%; N, 10.35%.

(*E*)-3-Phenyl-5-(4′-methoxyphenylamino)-2-styryl-1,3,4-thiadiazol-3-ium chloride (**5b**).

Red solid; yield 96%; mp 292–291 °C; FT-IR (ATR, υ cm^−1^): 3431 (N-H), 3043 (C-H), 2929 (C-H), 2717 (C=NH^+^), 1622 (C=C), 1595, 1575, 1454 (C=C), 1512 (C=N), 1342 (C-S), 1244 (C-O), 1109 (OCH_3_), 954 (C-H), 829, 773, 694 (C-H); ^1^H-NMR (500 MHz, DMSO-*d*_6_) δ 12.73 (s, 1H, N-H), 8.24 (d, *J* = 9.7 Hz, 2H, H-3′’, H-5′’), 8.09 (d, *J* = 16.0 Hz, 1H, H-7), 8.00 (d, *J* = 9.7 Hz, 2H, H-2′’, H-6′’), 7.83-7.75 (m, 6H, H-2′, H-3′, H-4′, H-5′, H-6′, H-4′’), 7.52 (d, *J* = 9.7 Hz, 2H, H-2′’’, H-6′’’), 7.24 (d, *J* = 16.0 Hz, 1H, H-6), 6.99 (d, *J* = 9.7 Hz, 2H, H-3′’’, H-5′’’), 3.73 (s, 3H, OCH_3_); ^13^C-NMR (125 MHz, DMSO-*d*_6_) δ 161.3 (C-2), 156.0 (C-5), 148.5 (C-4′’’), 144.2 (C-7), 140.0 (C-1′), 137.0 (C-1′’), 131.8 (C-4′’), 131.7 (C-1′’’), 130.2 (C-2′, C-6′), 130.0 (C-3′’, C-5′’), 126.1 (C-2′’, C-6′’), 124.1 (C-2′’’, C-6′’’), 120.5 (C-4′), 115.5 (C-6), 114.6 (C-3′’’, C-5′’’), 55.3 (OCH_3_). Elemental analysis found: C, 65.05%; H, 4.58%; N, 10.04%. Calculated for C_23_H_20_ClN_3_OS (MW 421.94): C, 65.47%; H, 4.78%; N, 9.96%.

(*E*)-3-Phenyl-5-(4′-chlorophenylamino)-2-styryl-1,3,4-thiadiazol-3-ium chloride (**5c**).

Orange solid; yield 97%; mp 284–282 °C; FT-IR (ATR, υ cm^−1^): 3433 (N-H); 2922 (C-H); 2586 (C=NH^+^); 1620 (C=C); 1562, 1492 (C=C); 1537 (C=N); 1249 (C-S); 1097 (C-H); 1033 (C-Cl); 817 (C-H); 761e 686 (C-H). ^1^H-NMR (500 MHz, DMSO-*d*_6_) δ 12.82 (s, 1H, N-H), 8.02 (d, *J* = 19.7 Hz, 1H, H-7), 7.84 (dd, *J* = 3.9 Hz, 2H, H-3′’, H-5′’), 7.75 ( m, 5H, H-2′’, H-6′’, H-4′’, H-3′, H-5′), 7.61 (d, *J* = 10.5 Hz, 2H, H-2′, H-6′), 7.47 (m, 5H, H-2′’’, H-6′’’, H-3′’’, H-5′’’, H-4′), 7.04 (d, *J* = 19.7 Hz, 1H, H-6); ^13^C-NMR (125 MHz, DMSO-*d*_6_) δ 163.2 (C-2), 158.8 (C-5), 148.2 (C-7), 137.4 (C-1′’’), 136.9 (C-1′), 133.8 (C-1′’), 132.0 (C-4′’), 131.7 (C-4′), 130.2 (C-3′, C-5′), 129.4 (C-3′’’, C-5′’’), 129.1 (C-2′’’, C-6′’’), 129.2 (C-2′, C-6′), 127.7 (C-4′’’), 126.2 (C-2′’, C-6′’), 120.1 (C-3′’, C-5′’), 111.4 (C-6). Elemental analysis found: C, 61.84%; H, 3.97%; N, 9.98%. Calculated for C_22_H_17_Cl_2_N_3_S (MW 426.36): C, 61.98%; H, 4.02%; N, 9.86%.

(*E*)-3-Phenyl-5-(4′-bromophenylamino)-2-styryl-1,3,4-thiadiazol-3-ium chloride (**5d**).

Orange solid; yield 96%; mp 298–296 °C; FT-IR (ATR, υ cm^−1^): 3432 (N-H); 3037 (C-H); 2663 (C=NH^+^); 1604 (C=C); 1560, 1484 e 1442 (C=C); 1540 (C=N); 1309 (C-S); 1114 (C-H); 948, 669 (C-Br); 827 (C-H); 755, 690 (C-H). ^1^H-NMR (500 MHz, DMSO-*d*_6_) δ 12.62 (s, 1H, N-H), 8.03 (d, *J* = 19.7 Hz, 1H, H-7), 7.83 (dd, *J* = 4.5 Hz, 2H, H-3′’, H-5′’), 7.75 (m, 5H, H-2′’, H-6′’, H-4′’, H-3′, H-5′), 7.60 (d, *J* = 12.1 Hz, 2H, H-2′, H-6′), 7.54 (d, *J* = 12.1 Hz, 2H, H-3′’’, H-5′’’), 7.47 (m, 5H, H-2′’’, H-6′’’, H-4′), 7.04 (d, *J* = 19.7 Hz, 1H, H-6); ^13^C-NMR (125 MHz, DMSO-*d*_6_) δ 163.4 (C-2), 158.9 (C-5), 148.2 (C-7), 137.9 (C-1′’’), 136.9 (C-1′), 133.8 (C-1′’), 132.3 (C-3′’’, C-5′’’), 132.0 (C-4′’), 131.8 (C-4′), 130.2 (C-3′, C-5′), 129.2 (C-2′’’, C-6′’’), 129.2 (C-2′, C-6′), 126.2 (C-2′’, C-6′’), 120.5 (C-3′’, C-5′’), 115.8 (C-4′’’), 111.5 (C-6). Elemental analysis found: C, 56.08%; H, 3.57%; N, 9.02%. Calculated for C_22_H_17_BrClN_3_S (MW 470.81): C, 56.12%; H, 3.64%; N, 8.92%.

### 3.6. Biological Assays

#### 3.6.1. Cell Viability Assays

The 1 × 10^5^ cells/mL were incubated with or without compounds **5a**, **5b**, **5c, 5d** or DMSO (Sigma-Aldrich/Merck, St. Louis, MO, USA) at 37 °C in humidified atmosphere with a 5% CO_2_. After 72 h, the cellular viability was assessed using 5 mg/mL of 3-(4,5-dimethylthiazol-2-yl)-2,5-diphenyltetrazolium bromide (MTT, Sigma-Aldrich/Merck). Cells were incubated for 3 h, centrifuged, and formazan crystal was dissolved with DMSO. Plates were read on a Paradigma^®^ spectrofluorometer (Molecular Devices, San Jose, CA, USA) at a 490 nm wavelength. Cell death was also evaluated using Propidium Iodide (Biolegend) exclusion after a 24 h culture under the same conditions described above. The fluorescence was examined by FACSCalibus flow cytometry (BD) and 10,000 events were acquired for the experimental data. Analysis was accomplished using Summit V4.0 or FlowJo software (Becton, Dickingson and Company, Santa Barbara, CA, USA).

#### 3.6.2. Reactive Oxygen Species (ROS) Detection

The 1 × 10^5^ cells/mL were incubated with or without compounds **5a–d** (12.5 µM) or DMSO for 30 min at 37 °C in a humidified atmosphere with a 5% CO_2_. Cells were washed with phosphate buffer (Sigma-Aldrich/Merck) and incubated with 50 µM of Dihydrorhodamine 123 (DHR; Sigma-Aldrich/Merck). After 15 min at 37 °C, cells were washed and the fluorescence was examined using FACSCalibus flow cytometry. A total of 10,000 events was acquired for the experimental data, and a gate based on forward and side scatter parameters was made to analyze only viable cells. Analysis was accomplished using Summit V4.0 or FlowJo software.

#### 3.6.3. DNA Interaction Assay

Fluorescence emission spectroscopic titration experiments of DNA were conducted in the absence and presence of a constant concentration of the mesoionic compounds **5a–d**. The assay was performed using 2 and 10 ng/mL of salmon DNA (Sigma-Aldrich/Merck) at 37 °C. After that, the fluorescence emission was recorded after 1 h of DNA incubation with or without compounds **5a–d** (25 µM) or DMSO, over a wavelength range of 300 to 600 nm in a Tris-acetate-EDTA buffer (Promega) at room temperature. The slit widths for excitation and emission were set to 5.0 nm. The fluorescence was examined using a F4500 fluorometer (Hitachi, New Jersey, NJ, USA).

#### 3.6.4. Statistical Analysis

Statistical analysis was performed by one-way analysis of variance (ANOVA) and means were compared using a Tukey post-test. The IC50 values were obtained using nonlinear regression. Data were analyzed using GraphPad Prism-5 software (GraphPad software, San Diego, CA, USA), and *p* ≤ 0.05 was considered statistically significant.

### 3.7. Spectroscopic HSA Binding Studies

The steady-state fluorescence spectra were measured in the 290–450 nm range, at 296, 303, and 310 K, with the excitation wavelength at λ = 295 nm. To a 3.0 mL solution containing an appropriate concentration of HSA (1.00 × 10^−5^ M), successive aliquots from a stock solution of each mesoionic under study (1.00 × 10^−3^ M in DMSO) were added, with final concentrations of 0.17, 0.33, 0.50, 0.66, 0.83, 0.99, 1.15, and 1.32 × 10^−5^ M. The addition was done manually by using a micro syringe. The inner filter correction for the observed steady-state fluorescence of HSA:mesoionic compounds was done in order to compensate for the absorption value of **5a–d** at the excitation and emission wavelengths (295 and 340 nm, respectively) when applying Equation (1) [50]:

(1) $$F_{\mathrm{cor}}=F_{\mathrm{obs}}10^{\left[\frac{A_{\mathrm{exc}}+A_{\mathrm{em}}}{2}\right]}$$

where *F_cor_* and *F_obs_* are the corrected and observed steady-state fluorescence intensity values, respectively, while *A_ex_* and *A_em_* represent absorbance values for each ligand at the excitation and emission wavelengths (λ = 295 and 340 nm, respectively).

The binding properties of each ligand toward HSA—Stern–Volmer quenching constant (*K_SV_*), bimolecular quenching rate constant (*k_q_*), modified Stern–Volmer binding constant (*K_a_*), enthalpy change (*ΔH*°), entropy change (*ΔS*°), and Gibbs’ free energy change (*ΔG*°)—were calculated from the steady-state fluorescence quenching data according to Equations (2)–(5) [47,48]:

(2) $$\frac{F_{0}}{F}=1+k_{q}\tau_{0}\left[Q\right]=1+K_{SV}\left[Q\right]$$

(3) $$\frac{F_{0}}{F_{0-F}}=\frac{1}{f\left[Q\right]K_{a}}+\frac{1}{f}$$

(4) $$\ln K_{a}=-\frac{\Delta H^{0}}{RT}+\frac{\Delta S^{0}}{R}$$

(5) $$\Delta G^{0}=\Delta H^{0}-T\Delta S^{0}$$

where *F_0_* and *F* are the steady-state fluorescence intensities of HSA in the absence and presence of mesoionics, respectively. [*Q*] and *τ_0_* are the ligand concentration and fluorescence lifetime, respectively, of the fluorophore in the absence of quencher (5.54 × 10^−9^ s—experimental data, see Section 2.3.1). T, *ƒ*, and *R* are the temperature (296, 303, and 310 K), fraction of the initial fluorescence intensity that corresponds to fluorophore that is accessible to the quencher (*f* ≈ 1.0), and gas constant (8.3145 J/mol.K), respectively.

The time-resolved fluorescence decay of a 3.0 mL solution of HSA (1.00 × 10^−5^ M, in phosphate buffer solution-PBS) was measured in the absence and presence of the maximum concentration of each mesoionic used in the steady-state fluorescence studies (1.32 × 10^−5^ M) at room temperature (ca 298 K).

The CD spectra were recorded in the 200–250 nm range at 310 K for free serum albumin (1.00 × 10^−6^ M) and for HSA with the higher concentration of each mesoionic used in the steady-state fluorescence studies (1.32 × 10^−5^ M). Each spectrum was taken on the average of three scans. The obtained data were further converted into mean residue ellipticity (MRE) according to Equation (6) [50]:

(6) $$MRE=\frac{\theta }{\left(10.n.l.C_{p}\right)}$$

where *θ* is the observed ellipticity (mdeg); *n* is the number of amino acid residues (585 to HSA) [45], *l* is the length of the optical cuvette (1.0 cm), and *C_p_* is the molar concentration of HSA (1.00 × 10^−6^ M). The **5a–d** induced helical content perturbation at 208 and 222 nm were calculated using Equation (7) [46]:

(7) $$(A)\;\alpha -helix\%\frac{\left(-MRE_{208}-4000\right)}{\left(33000-4000\right)}\times 100\;(B)\;\alpha -helix\%\frac{\left(-MRE_{222}-2340\right)}{\left(30300\right)}\times 100$$

where *MRE*_208_ and *MRE*_222_ are the significant molar residual ellipticities (deg.cm^2^/dmol) at 208 nm and 222 nm, respectively.

### 3.8. Zeta Potential Measurements for HSA Surface Perturbation

All zeta potential (ZP) measurements were performed with 10 runs at 298 K and results were reported in terms of ZP ± SD. The ZP was measured for the HSA solution (1.0 × 10^−5^ M in PBS solution) without and in the presence of the mesoionic compounds at the maximum concentration used in the steady-state fluorescence experiments (1.32 × 10^−5^ M) at room temperature (ca. 298 K).

### 3.9. Molecular Docking Procedure for DNA and HSA

The chemical structure for the mesoionic compounds (**5a–d**) was built and energy-minimized with Density Functional Theory (DFT), with the Becke-3-Lee Yang Parr (B3LYP) method with the standard 6-31G* basis set, available in Spartan’18 software (Wavefunction, Inc., Irvine, CA, USA) [79]. The crystallographic structures for the DNA and HSA were obtained from the Protein Data Bank (PDB) with access codes 1BNA [80] and 1N5U [45], respectively. Molecular docking was performed with GOLD 5.7 software (CCDC) [81]. Hydrogen atoms were added to the macromolecules according to the data inferred by the GOLD 5.7 software (Cambridge Crystallographic Data Centre, Cambridge, CB2 1EZ, UK) on the ionization and tautomeric states. For DNA, a 10 Å radius spherical cavity around minor and major groove was defined as the binding site for the molecular docking calculations and for has, and a 10 Å radius spherical cavity around subdomains IIA, IIIA, and IB was defined. The scoring function used was “*ChemPLP*”, which is the standard function of GOLD 5.7 software. The figures of the docking poses for the largest docking score value were generated with the PyMOL Delano Scientific LLC program [82]. For more details, see previous publications [46,47,48].

## 4. Conclusions

The results obtained in this work show new promising chemotherapy agents against MT2 and C92 cell lines infected with HTLV-1, which causes adult T-cell leukemia/lymphoma (ATLL), and in uninfected Jurkat cells with IC_50_ values lower the 10 μM. The compounds containing electron donor substituents (R=CH_3_ or OCH_3_) were more active and with lower hydrophobicity. Furthermore, the compounds showed cell death by necrosis and DNA interaction, especially those containing electron donor substituents.

The interaction between HSA and the mesoionic compounds is spontaneous and occurs via a ground-state association (static fluorescence quenching mechanism). The binding is moderate (*K_a_* ~ 10^4^ M^−1^), being enthalpically and entropically driven for HSA:**5a** and HSA:**5b**, while for the other mesoionic compounds, i.e., **5c** and **5d**, is only entropically driven. The binding does not significantly perturb both the secondary and surface structures of the albumin, and the main binding pocket is site I (subdomain IIA), where Trp-214 residue can be found. Overall, all the mesoionic compounds presented good binding parameters toward serum albumin, indicating feasible biodistribution in the human bloodstream.

## Figures and Tables

**Table 1:** In vitro anti-cancer activity results for the mesoionic compounds **5a–d.**

Cell Lines	IC_50_ Values for the Mesoionic Compounds 5a–d (μM)			
	5a	5b	5c	5d
MT2	1.51	5.09	6.15	7.70
C91/PL	2.82	4.50	4.50	5.25
Jurkat	2.15	1.74	5.60	2.27

IC_50_ = drug concentration required to inhibit the cell viability by 50% after 72 h of incubation.

**Table 2:** Binding constant values for the interaction between human serum albumin (HSA) and the mesoionic compounds **5a–d.**

Sample	T (K)	*K_SV_* (× 10^5^) (M^−1^)	*k_q_* (× 10^13^) (M^−1^s^−1^)	*K_a_* (× 10^4^) (M^−1^)	*ΔH°* (kJ.mol^−1^)	*ΔS°* (× 10^−2^) (kJ.mol^−1^K^−1^)	*ΔG°* (kJ.mol^−1^)
	296	1.19 ± 0.03	2.15	7.68 ± 0.26			−27.7
**HSA:5a**	303	1.20 ± 0.03	2.16	7.51 ± 0.26	−2.64 ± 0.15	8.46 ± 0.05	−28.3
	310	1.21 ± 0.03	2.18	7.32 ± 0.26			−28.9
	296	1.10 ± 0.02	1.99	8.57 ± 0.26			−28.0
**HSA:5b**	303	1.14 ± 0.03	2.06	8.44 ± 0.26	−1.88 ± 0.17	8.81 ± 0.06	−28.6
	310	1.15 ± 0.03	2.07	8.27 ± 0.26			−29.2
	296	1.30 ± 0.03	2.35	8.53 ± 0.26			−28.1
**HSA:5c**	303	1.36 ± 0.04	2.45	9.06 ± 0.26	6.27 ± 0.10	11.6 ± 0.1	−28.9
	310	1.40 ± 0.03	2.53	9.57 ± 0.26			−29.7
	296	1.45 ± 0.02	2.61	8.71 ± 0.26			−27.9
**HSA:5d**	303	1.54 ± 0.03	2.79	9.02 ± 0.26	3.43 ± 0.16	10.6 ± 0.1	−28.7
	310	1.60 ± 0.03	2.89	9.28 ± 0.26			−29.4

r^2^ for *K_SV_* and *k_q_*: 0.9986–0.9999; r^2^ for *K_a_*: 0.9961–0.9998; r^2^ for *ΔH°, ΔS°* and *ΔG°*: 0.9920–0.9979.

**Table 3:** Time-resolved fluorescence parameters for the interaction between HSA and the mesoionic compounds **5a–d.**

Sample	*τ_1_* (ns)	Relative %	*τ_2_* (ns)	Relative %	χ^2^
**HSA**	1.73 ± 0.16	20.7	5.54 ± 0.12	79.3	1.095
**HSA:5a**	1.61 ± 0.13	26.4	5.38 ± 0.13	73.6	1.140
**HSA:5b**	1.66 ± 0.12	23.9	5.25 ± 0.11	76.1	1.106
**HSA:5c**	1.68 ± 0.11	33.0	5.66 ± 0.16	67.0	1.155
**HSA:5d**	1.65 ± 0.10	29.8	5.37 ± 0.13	70.2	1.038

**Table 4:** α-helix % values at 208 nm and 222 nm (Circular dichroism spectroscopy (CD) measurements) and ζ, conductance and current values (zeta potential (ZP) measurements) for HSA without and in the presence of mesoionic compounds **5a–d** at 310 K.

	CD Results		ZP Results		
Sample	208 nm	222 nm	ζ (mV)	Conductance (µs)	Current (mA)
**HSA**	86.7%	80.1%	−9.15 ± 1.79	29,645	150
**HSA:5a**	84.0%	78.5%	−8.62 ± 1.40	29,615	150
**HSA:5b**	82.4%	77.3%	−8.55 ± 1.46	29,642	150
**HSA:5c**	83.8%	78.8%	−8.77 ± 1.52	29,622	150
**HSA:5d**	82.0%	77.7%	−8.69 ± 1.50	29,638	150

**Table 5:** Molecular docking score values (dimensionless) for the interaction between DNA:**5a–d** and HSA:**5a–d**.

	DNA		HSA		
Compound	Minor Groove	Major Groove	Site I	Site II	Site III
**5a**	83.9	11.2	78.3	40.2	39.5
**5b**	84.8	10.6	80.1	40.0	38.1
**5c**	83.4	13.8	86.2	45.6	41.1
**5d**	87.4	13.6	85.0	42.9	40.8

## Supplementary Materials

The following are available online at https://www.mdpi.com/1420-3049/25/11/2537/s1, Figures S1–S4: FTIR, 1H-NMR and 13C-NMR spectra for **5a**, **5b**, **5c**, and **5d**, respectively. Figure S5: Fluorescence emission spectra for **5a**, **5c**, and **5d** (25 μM) in the presence of 100 ng/mL of DNA. Figure S6: Steady-state fluorescence quenching for HSA without and in the presence of **5a**, **5b**, **5c**, or **5d** at 310 K. Figure S7: Stern–Volmer plots for the interaction between HSA and the mesoionic compounds at 296, 303, and 310 K. Figure S8: Time-resolved fluorescence decay for the interaction between HSA and the mesoionic compounds **5a–d** in a PBS solution. [HSA] = 1.00 × 10^−5^ M and [mesoionic] = 1.32 × 10^−5^ M. IRF is the instrument response factor. Figure S9: Modified Stern–Volmer plots for the interaction between HSA and the mesoionic compounds at 296, 303, and 310 K. Figure S10: Van’t Hoff plot for the interaction between HSA and the mesoionic compounds. Figure S11: Circular dichroism spectra for HSA without and in the presence of **5a**, **5b**, **5c**, or **5d** at 310 K.
