# Supplementary material for: Synthetic (E)-3-Phenyl-5-(phenylamino)-2-styryl-1,3,4-thiadiazol-3-ium Chloride Derivatives as Promising Chemotherapy Agents on Cell Lines Infected with HTLV-1
Source: Molecules. 2020 May 29;25(11):2537. doi: 10.3390/molecules25112537 (PMC7321218; doi:10.3390/molecules25112537)
Supplement: Supplementary file 1 [file molecules-25-02537-s001.pdf]

Synthetic (*E*)-3-phenyl-5-(phenylamino)-2-styryl-1,3,4-thiadiazol-3-ium chloride derivatives as promising chemotherapy agents on cell lines infected with HTLV-1

Danilo Sousa-Pereira [1], Thais Silva de Oliveira [2], Rojane O. Paiva [2], Otávio Augusto Chaves [1,3], José C. Netto-Ferreira [1,4*], Juliana Echevarria-Lima [2*], Aurea Echevarria [1*]

[1] Instituto de Química, Universidade Federal Rural do Rio de Janeiro, Seropédica, Rio de Janeiro, 23.890-000, Brazil; sousadanilo90@gmail.com (D.S.-P.); echevarr@ufrrj.br (A.E.)

[2] Laboratório de Imunologia Básica e Aplicada, Departamento de Imunologia, Instituto de Microbiologia Paulo de Góes, Universidade Federal do Rio de Janeiro, Rio de Janeiro, 21.941-590, Brazil; thais.silvadeoliveira@yahoo.com.br (T.S.O.); rojanedeoliveirapaiva@gmail.com (R.O.P.); juechevarria@micro.ufrj.br (J.E.-L.)

[3] Instituto SENAI de Inovação em Química Verde (ISI QV), Maracanã, Rio de Janeiro, 20.271-030, Brazil; otavioaugustochaves@gmail.com (O.A.C.)

[4] Qualidade e Tecnologia (INMETRO), Divisão de Metrologia Química, Instituto Nacional de Metrologia, Duque de Caxias, Rio de Janeiro, 25.250-020, Brazil; jcnetto.ufrrj@gmail.com (J.C.N-F.)

Supplementary Material

Index

Figure S1. FTIR, ^{1}H NMR and ^{13}C NMR (DEPT-Q) spectra of (*E*)-3-phenyl-5-(4′-methylphenylamino)-2-styryl-1,3,4-thiadiazol-3-ium chloride (**5a**). p.3

Figure S2. FTIR, ^{1}H NMR and ^{13}C NMR (DEPT-Q) spectra of (*E*)-3-phenyl-5-(4′-methoxyphenylamino)-2-styryl-1,3,4-thiadiazol-3-ium chloride (**5b**). p.4

Figure S3. FTIR, ^{1}H NMR and ^{13}C NMR (DEPT-Q) spectra of (*E*)-3-phenyl-5-(4′-chlorophenylamino)-2-styryl-1,3,4-thiadiazol-3-ium chloride (**5c**). p.5

Figure S4. FTIR, ^{1}H NMR and ^{13}C NMR (DEPT-Q) spectra of (*E*)-3-phenyl-5-(4′-bromophenylamino)-2-styryl-1,3,4-thiadiazol-3-ium chloride (**5d**). p.6

Figure S5. Fluorescence emission spectra of **5a** ,**5c** and **5d** (25 μM) in the presence of 100 ng/mL of DNA. A.U. = Arbitrary Unit. p.7

Figure S6. Steady-state fluorescence emission spectra for HSA and its quenching upon successive additions of (A) **5a**, (B) **5b**, (C) **5c**, and (D) **5d** at pH 7.4 and 310K. [HSA] = 1.00 × 10^{-5} M and [mesoionic compounds] = 0.17; 0.33; 0.50; 0.66; 0.83; 0.99; 1.15 and 1.32 × 10^{-5} M. p.8

Figure S7. Stern-Volmer plots for the interaction (A) HSA:**5a**, (B) HSA:**5b**, (C) HSA:**5c**, and (D) HSA:**5d** at 296, 303, and 310K. [HSA] = 1.00 × 10^{-5} M and [mesoionic compounds] = 0.17; 0.33; 0.50; 0.66; 0.83; 0.99; 1.15 and 1.32 × 10^{-5} M. p.9

Figure S8. Time-resolved fluorescence decay for the interaction between HSA and the mesoionic compounds **5a-d** in a PBS solution. [HSA] = 1.00 × 10^{-5} M and [mesoionic compounds] = 1.32 × 10^{-5} M. IRF is the instrument response factor. p.10

Figure S9. Modified Stern-Volmer plots for the interaction (A) HSA:**5a**, (B) HSA:**5b**, (C) HSA:**5c**, and (D) HSA:**5d** at 296, 303, and 310K. [HSA] = 1.00 × 10^{-5} M and [mesoionic compounds] = 0.17; 0.33; 0.50; 0.66; 0.83; 0.99; 1.15 and 1.32 × 10^{-5} M. p.11

Figure S10. Van't Hoff plot for the interaction (A) HSA:**5a**, (B) HSA:**5b**, (C) HSA:**5c**, and (D) HSA:**5d** at 296, 303, and 310K. p.12

Figure S11. Circular dichroism spectra for (A) HSA:**5a**, (B) HSA:**5b**, (C) HSA:**5c**, and (D) HSA:**5d** at 310K. [HSA] = 1.00 × 10^{-5} M and [mesoionics] = 1.32 × 10^{-5} M. p.13

FTIR

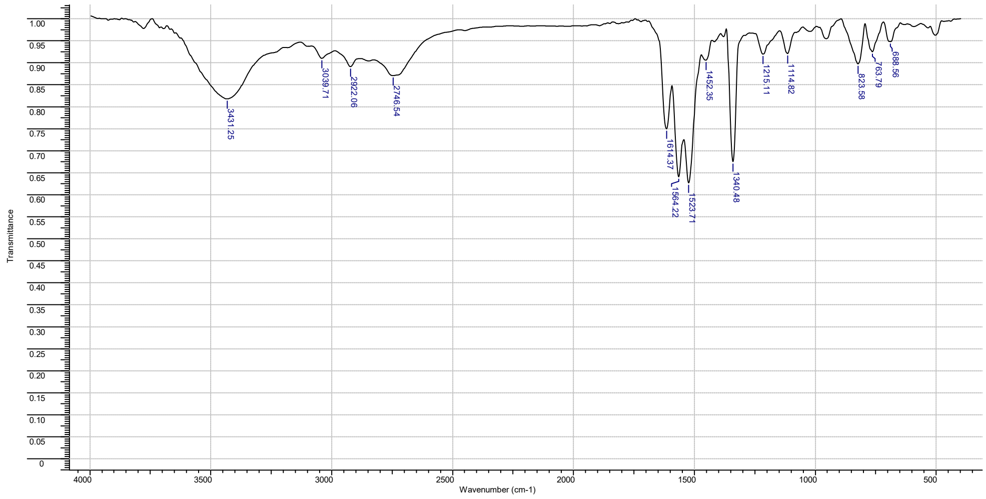

^{1}H NMR

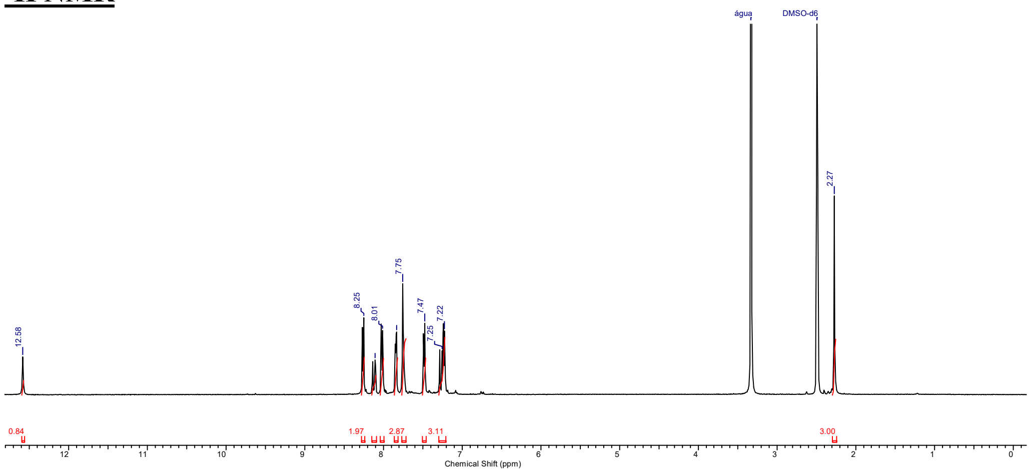

^{13}C NMR

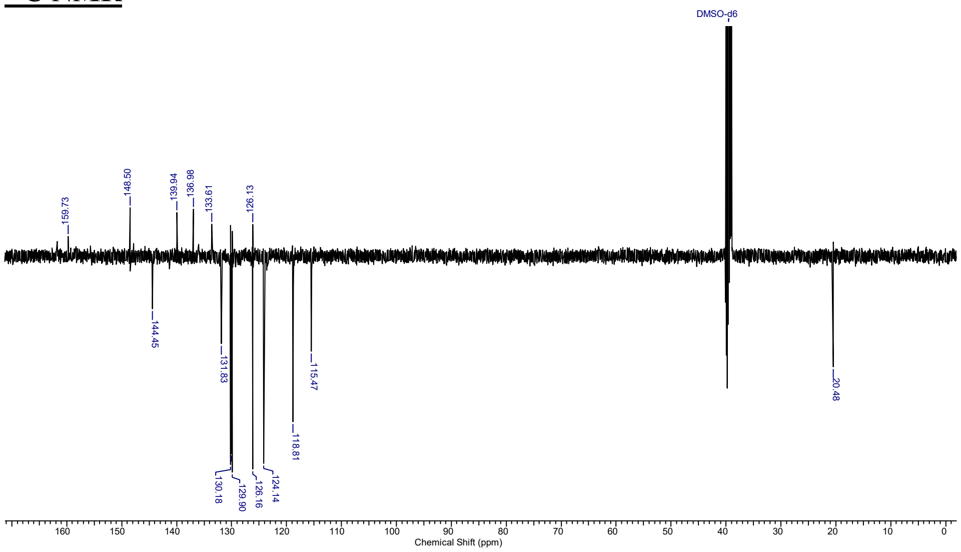

Figure S1. FTIR, ^{1}H NMR and ^{13}C NMR (DEPT-Q) spectra of (*E*)-3-phenyl-5-(4′-methylphenylamino)-2-styryl-1,3,4-thiadiazol-3-ium chloride (**5a**).

FTIR

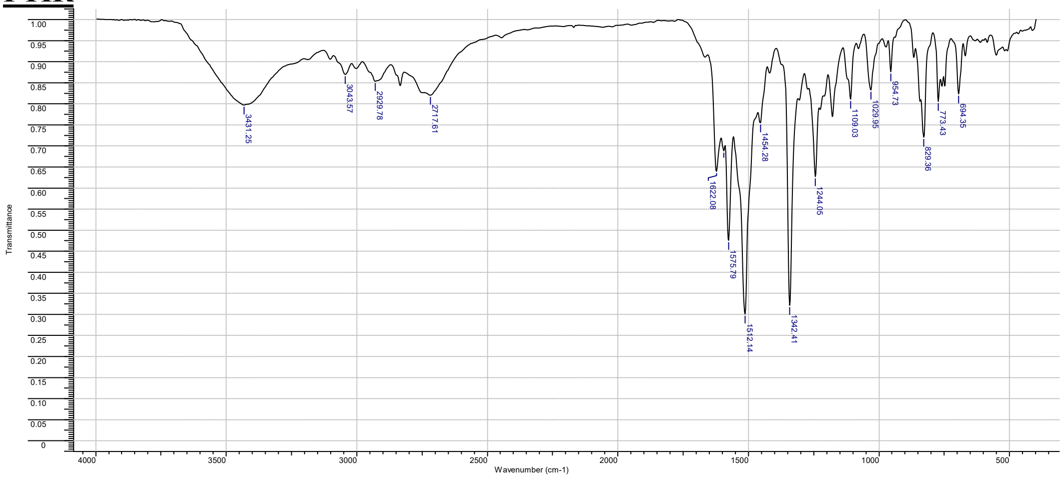

^{1}H NMR

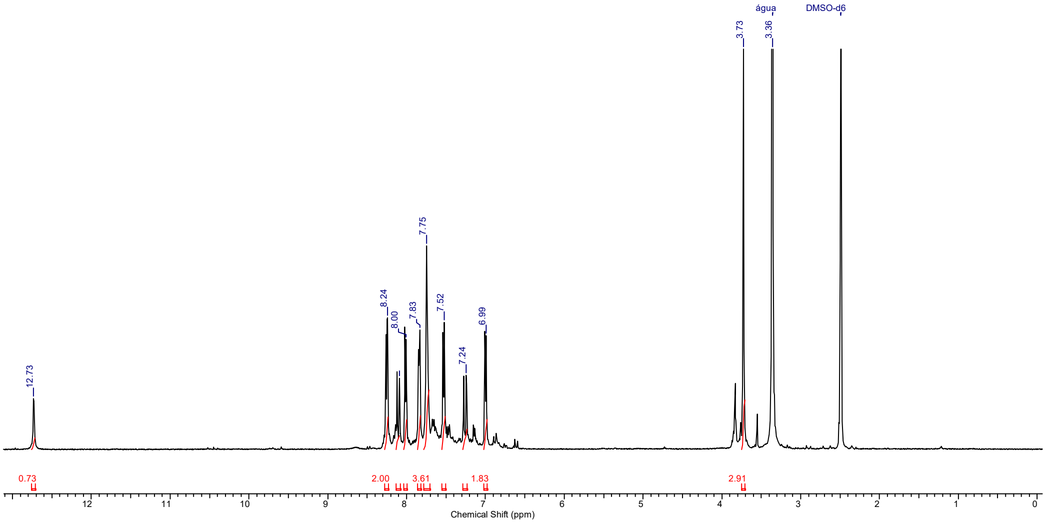

^{13}C NMR

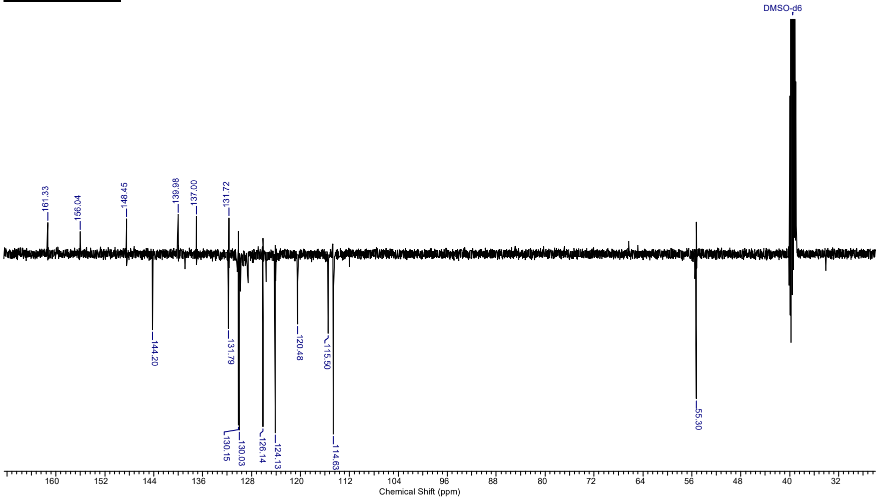

Figure S2. FTIR, ^{1}H NMR and ^{13}C NMR (DEPT-Q) spectra of (*E*)-3-phenyl-5-(4′-methoxyphenylamino)-2-styryl-1,3,4-thiadiazol-3-ium chloride (**5b**).

FTIR

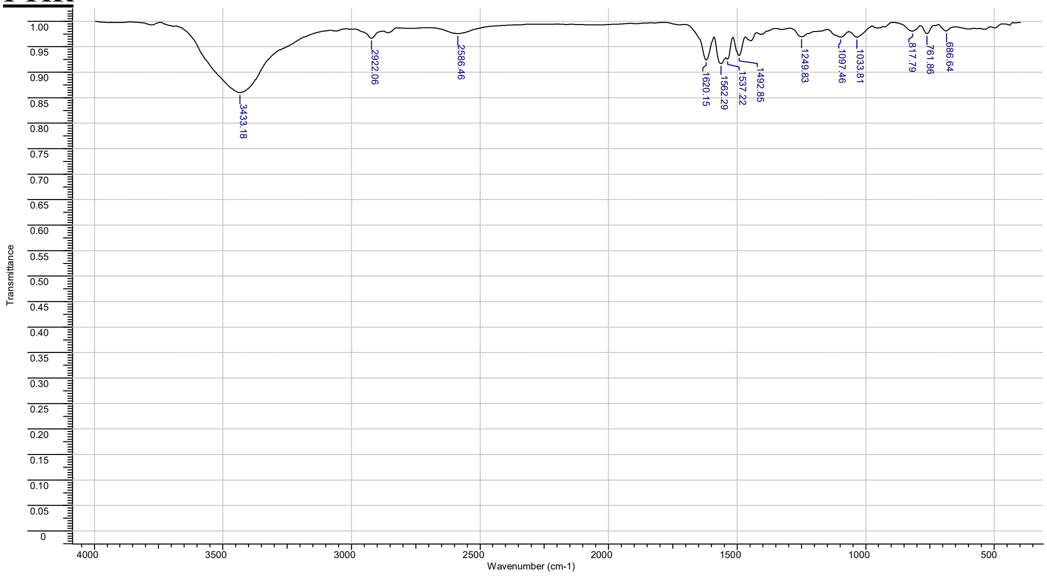

^{1}H NMR

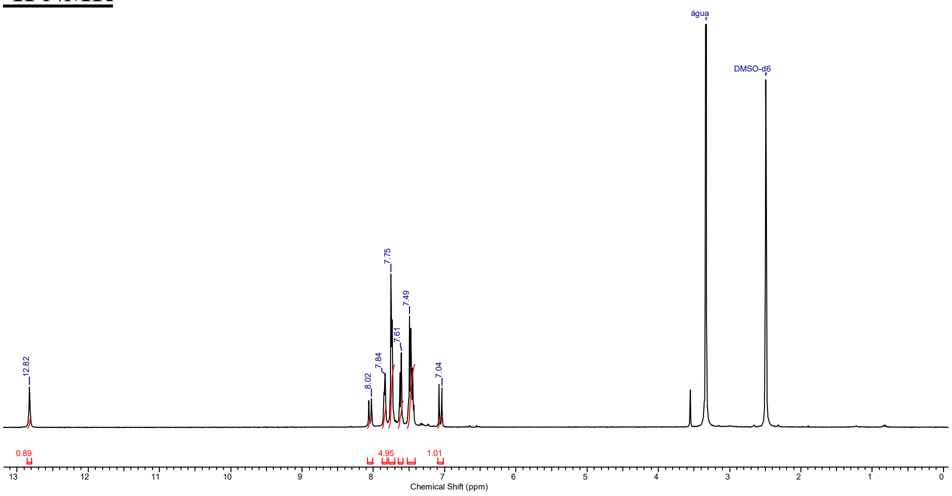

^{13}C NMR

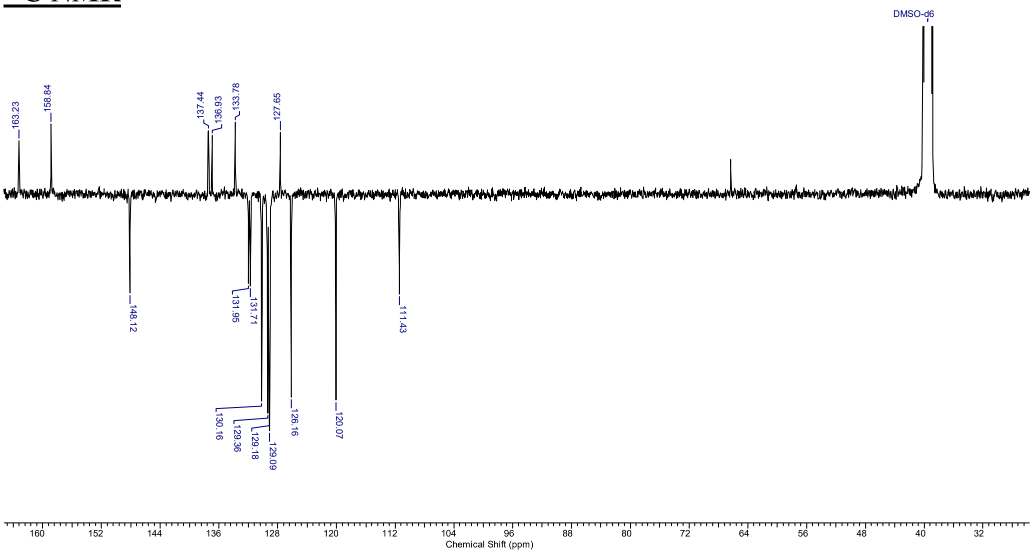

Figure S3. FTIR, ^{1}H NMR and ^{13}C NMR (DEPT-Q) spectra of (*E*)-3-phenyl-5-(4′-chlorophenylamino)-2-styryl-1,3,4-thiadiazol-3-ium chloride (**5c**).

FTIR

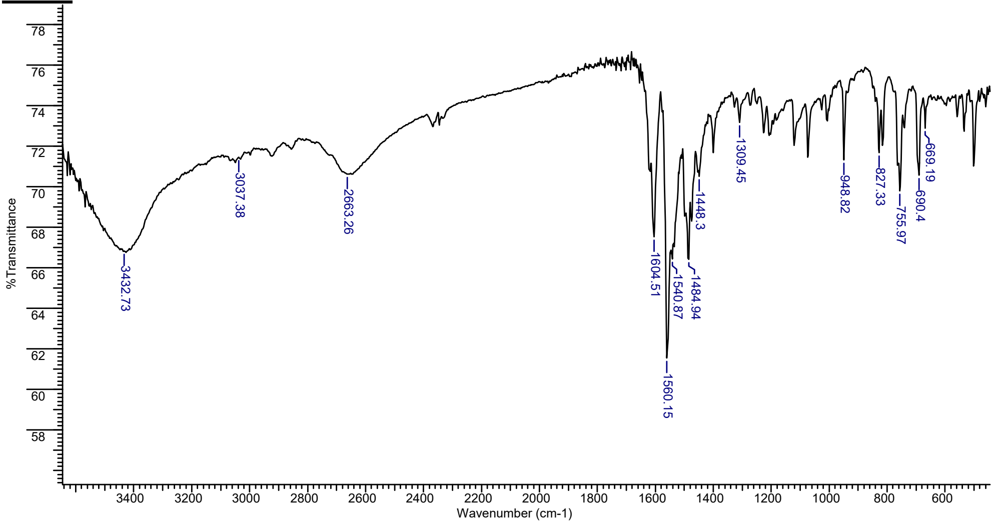

^{1}H NMR

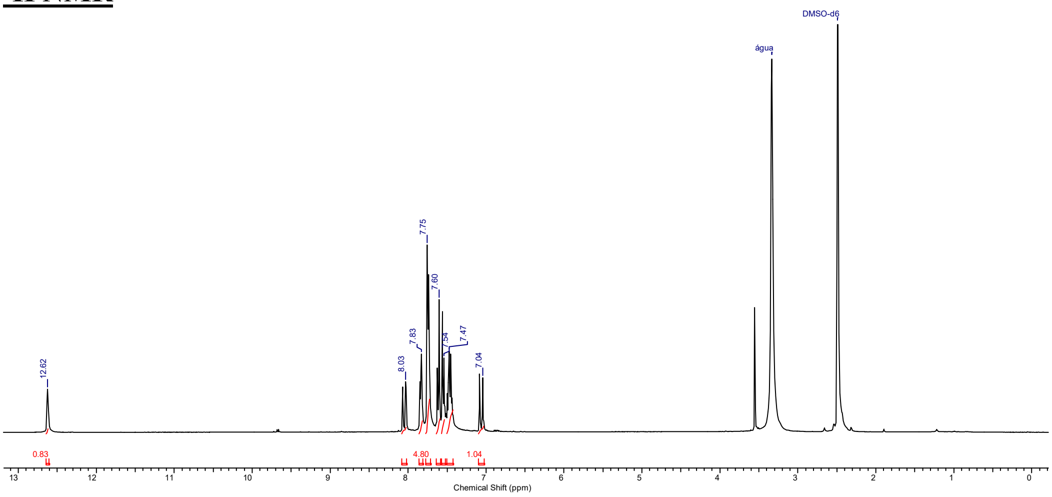

^{13}C NMR

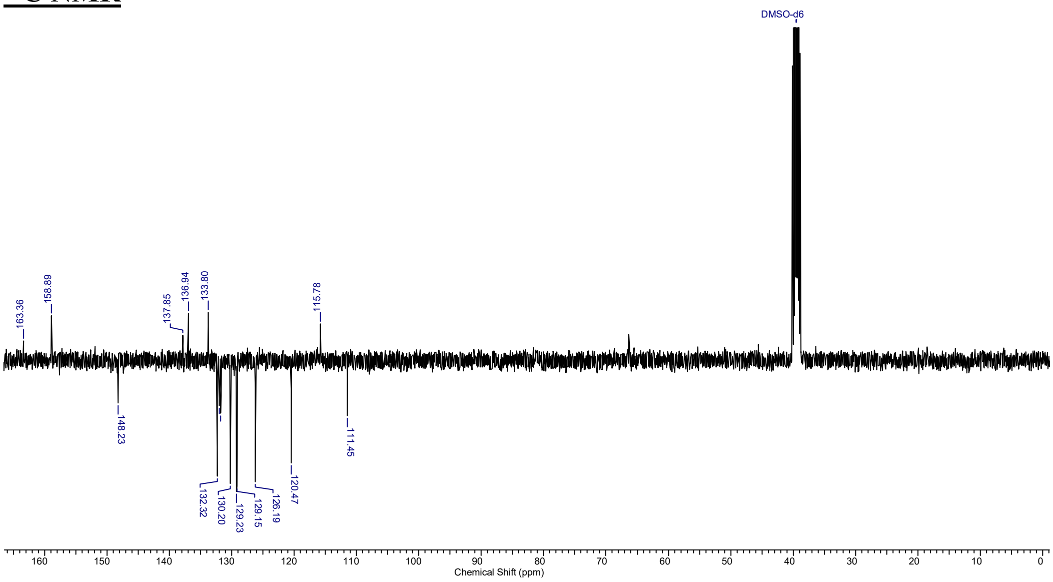

Figure S4. FTIR, ^{1}H NMR and ^{13}C NMR (DEPT-Q) spectra of (*E*)-3-phenyl-5-(4′-bromophenylamino)-2-styryl-1,3,4-thiadiazol-3-ium chloride (**5d**).

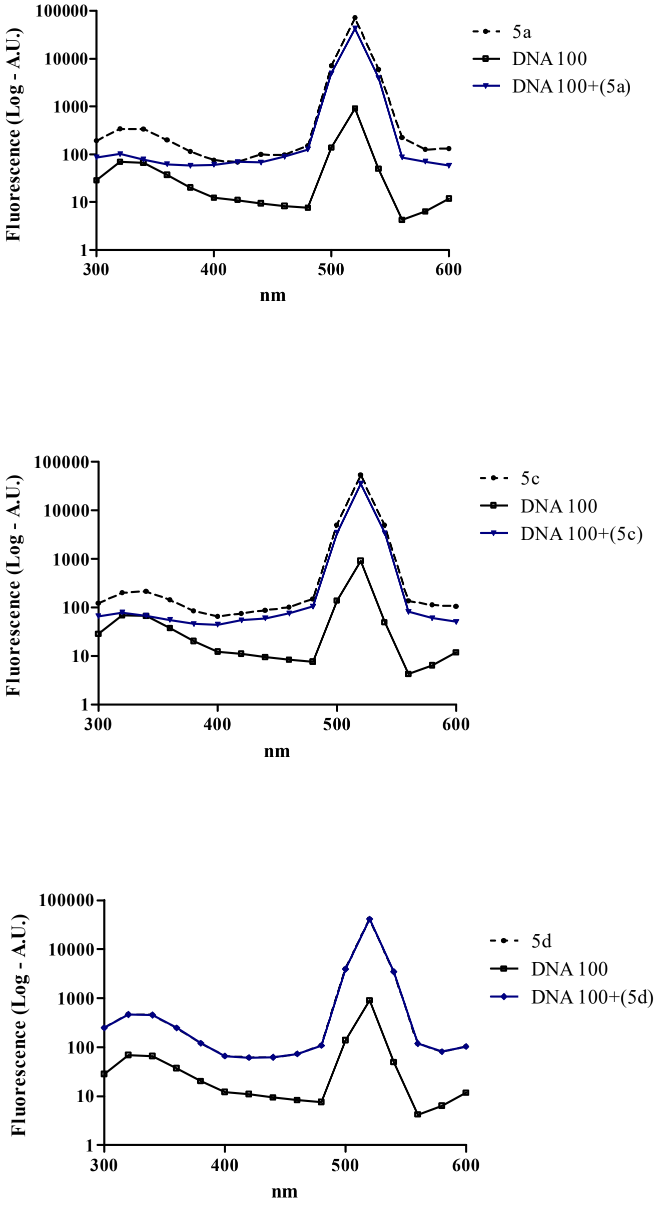

Figure S5. Fluorescence emission spectra of **5a** ,**5c** and **5d** (25 μM) in the presence of 100 ng/mL of DNA. A.U. = Arbitrary Unit.

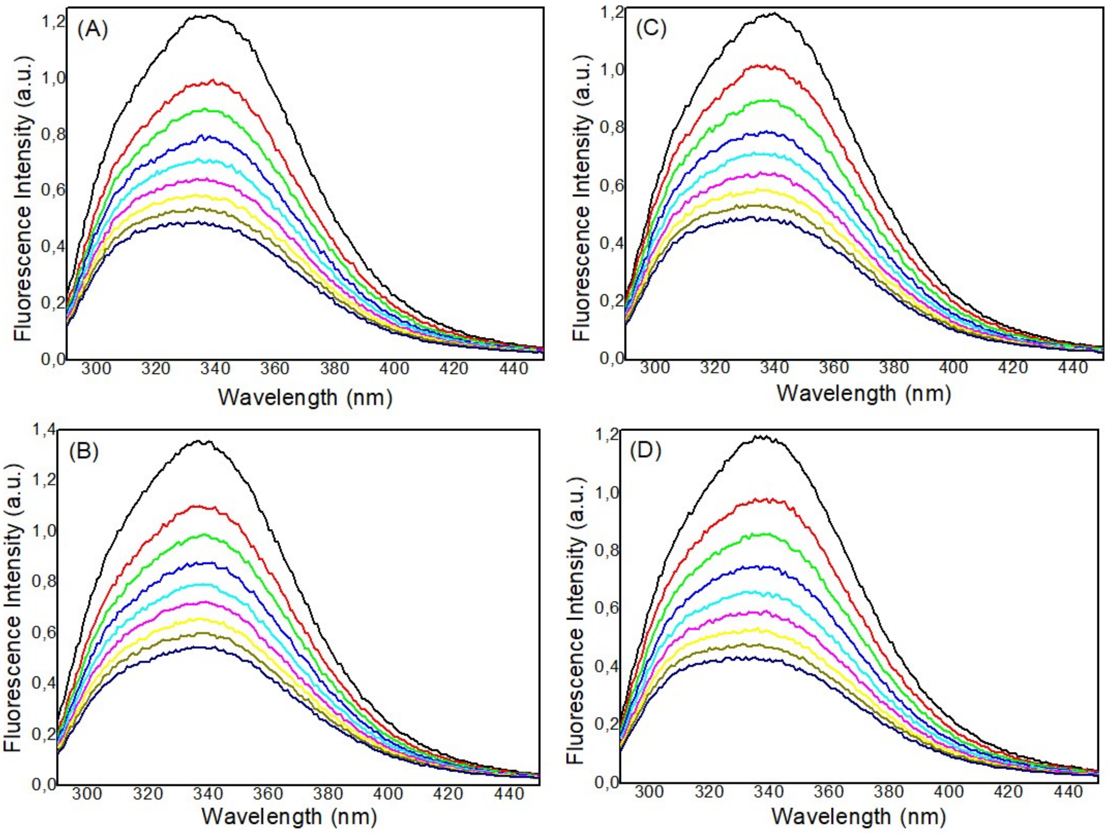

Figure S6. Steady-state fluorescence emission spectra for HSA and its quenching upon successive additions of (A) **5a**, (B) **5b**, (C) **5c**, and (D) **5d** at pH 7.4 and 310K. [HSA] = 1.00 × 10^{-5} M and [mesoionic compounds] = 0.17; 0.33; 0.50; 0.66; 0.83; 0.99; 1.15 and 1.32 × 10^{-5} M.

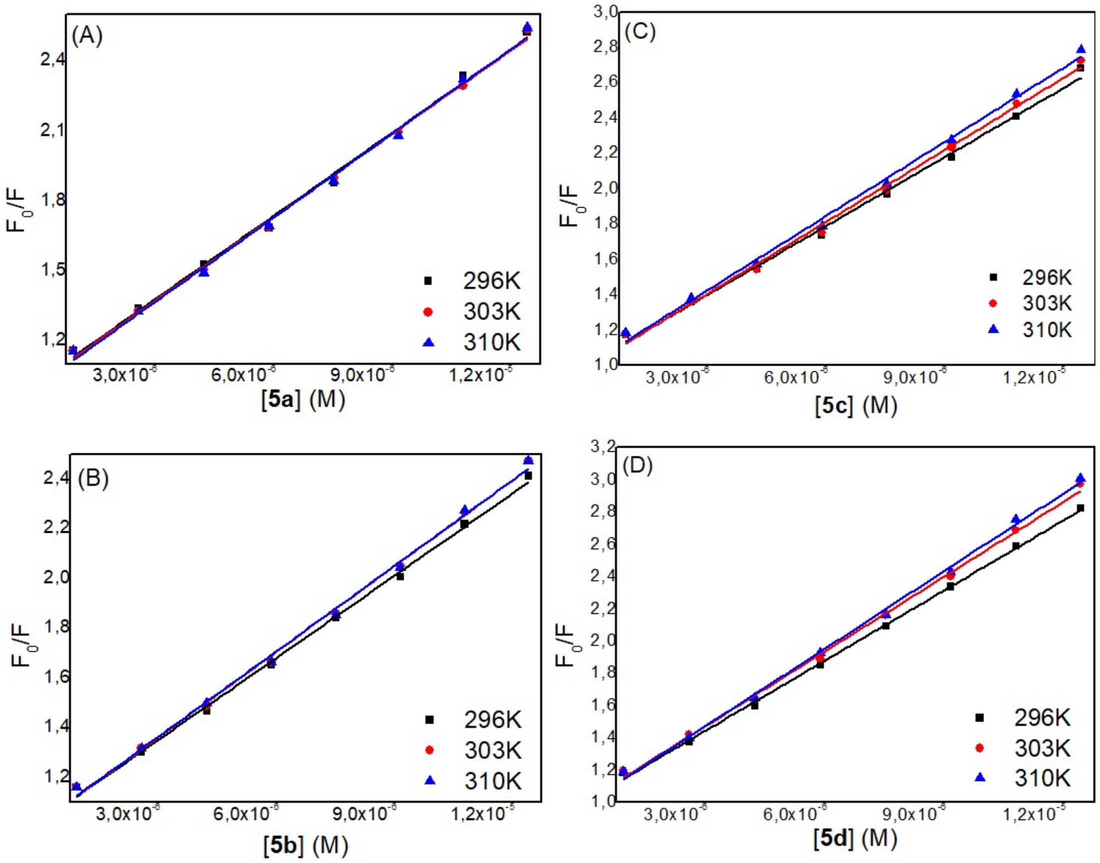

Figure S7. Stern-Volmer plots for the interaction (A) HSA:**5a**, (B) HSA:**5b**, (C) HSA:**5c**, and (D) HSA:**5d** at 296, 303, and 310K. [HSA] = 1.00 × 10^{-5} M and [mesoionic compounds] = 0.17; 0.33; 0.50; 0.66; 0.83; 0.99; 1.15 and 1.32 × 10^{-5} M.

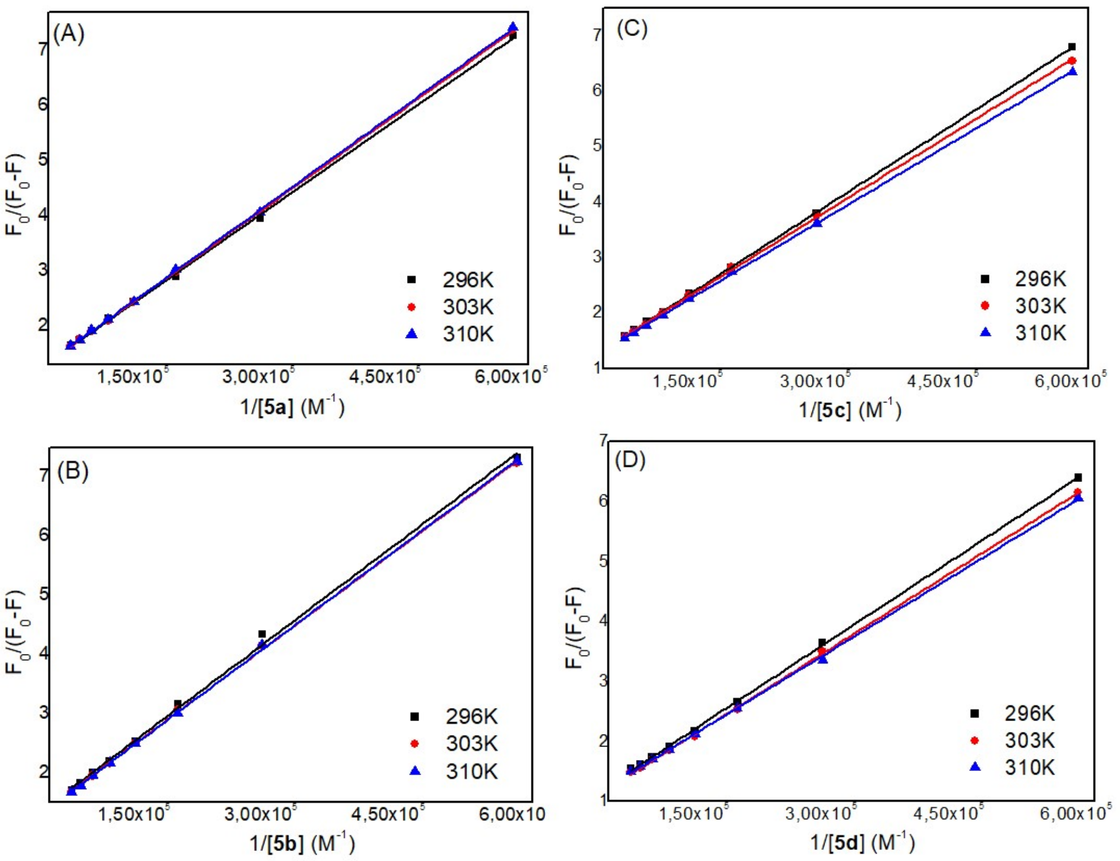

Figure S8. Modified Stern-Volmer plots for the interaction (A) HSA:**5a**, (B) HSA:**5b**, (C) HSA:**5c**, and (D) HSA:**5d** at 296, 303, and 310K. [HSA] = 1.00 × 10^{-5} M and [mesoionic compounds] = 0.17; 0.33; 0.50; 0.66; 0.83; 0.99; 1.15 and 1.32 × 10^{-5} M.

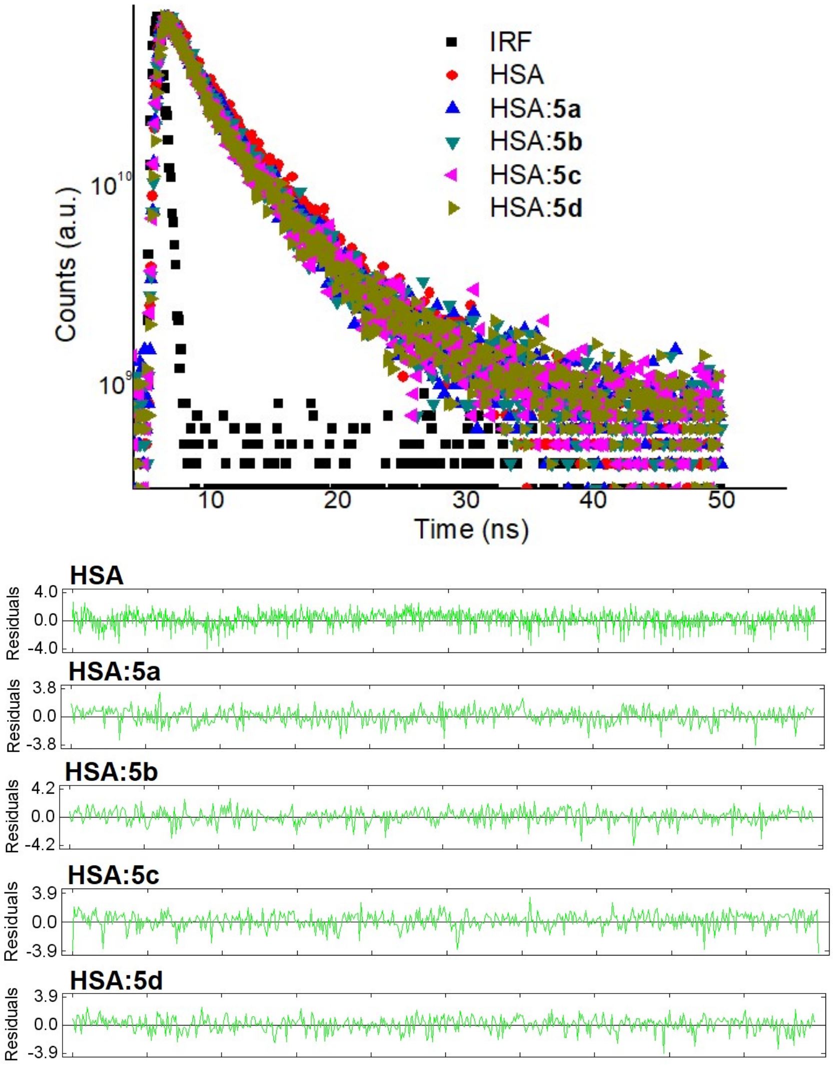

Figure S9. Time-resolved fluorescence decay for the interaction between HSA and the mesoionic compounds **5a-d** in a PBS solution. [HSA] = 1.00 × 10^{-5} M and [mesoionic compounds] = 1.32 × 10^{-5} M. IRF is the instrument response factor.

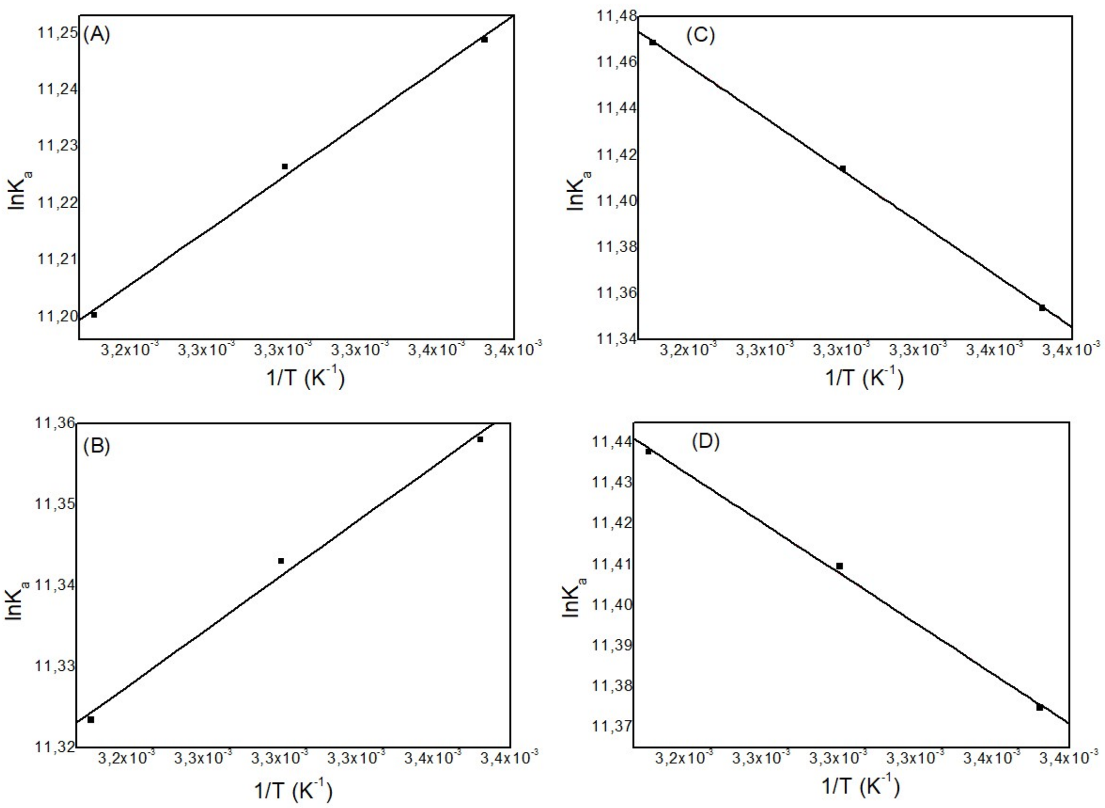

Figure S10. Van't Hoff plot for the interaction (A) HSA:**5a**, (B) HSA:**5b**, (C) HSA:**5c**, and (D) HSA:**5d** at 296, 303, and 310K.

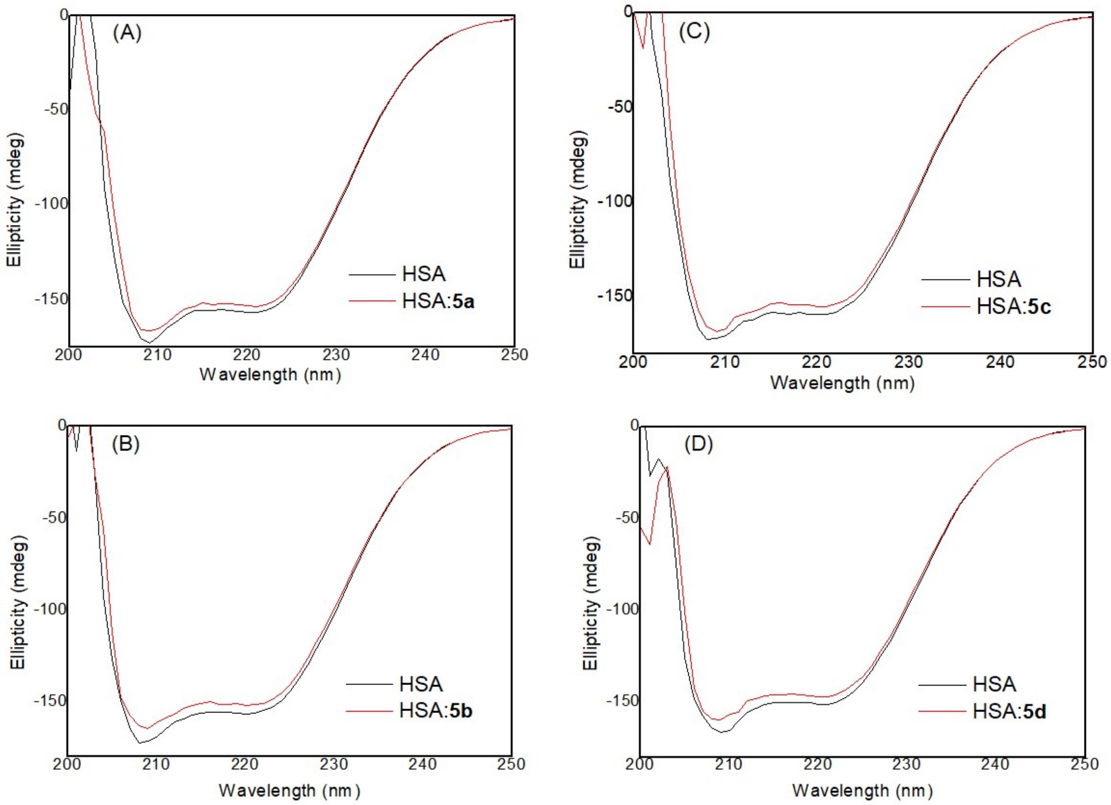

Figure S11. Circular dichroism spectra for (A) HSA:**5a**, (B) HSA:**5b**, (C) HSA:**5c**, and (D) HSA:**5d** at 310K. [HSA] = 1.00×10^{-5} M and [mesoionic compounds] = 1.32×10^{-5} M.
